# Measurements of Surrogate Respiratory Sessile Droplet pH and Implications for Exhaled Respiratory Aerosol and Airborne Disease Transmission

**DOI:** 10.1021/acscentsci.5c00284

**Published:** 2025-06-02

**Authors:** Jianghan Tian, Beiping Luo, Aidan Rafferty, Allen E. Haddrell, Ulrich K. Krieger, Jonathan P. Reid

**Affiliations:** † School of Chemistry, 1980 University of Bristol, Bristol BS8 1TS, United Kingdom; ‡ Institute for Atmospheric and Climate Science, ETH Zürich, CH-8092 Zürich, Switzerland; § Physical and Theoretical Chemistry Laboratory, University of Oxford, South Parks Road, Oxford OX1 3QZ, United Kingdom

## Abstract

Respiratory aerosol pH has been proposed as a key factor driving the infectivity loss of SARS-CoV-2 viruses and influenza A virus in exhaled aerosols, thus affecting the airborne transmission of respiratory diseases. Sodium bicarbonate acts as a principal buffer in biological systems, regulating blood pH and the CO_2_ balance between gas and liquid phases. Upon exhalation, changes in gas-phase conditions alter aerosol composition and pH. Despite Raman spectroscopy being used to quantify atmospherically relevant aerosol pH, the kinetics of CO_2_ partitioning and pH variability in respiratory droplets remain poorly understood. In this paper, a method to investigate the HCO_3_^–^/CO_3_^2–^ equilibrium in a surrogate respiratory fluid system within sessile droplets is proposed to elucidate the pH evolution of an exhaled respiratory aerosol. The enzymatic catalysis of CO_2_ hydration and H_2_CO_3_ dehydration is explored. Experimental results were used to benchmark the ResAM model, which simulates respiratory aerosol droplet thermodynamics and pH evolution. Simulated pH evolution profiles of picoliter droplets show size independence. Simulations for both sessile droplets and respiratory aerosols show that carbonic anhydrase significantly increases the rate of pH increase, and gas-phase CO_2_ levels are important for determining the final droplet pH. Consequences for understanding the aerobiological pathways for virus transmission are considered.

## Graphical abstract

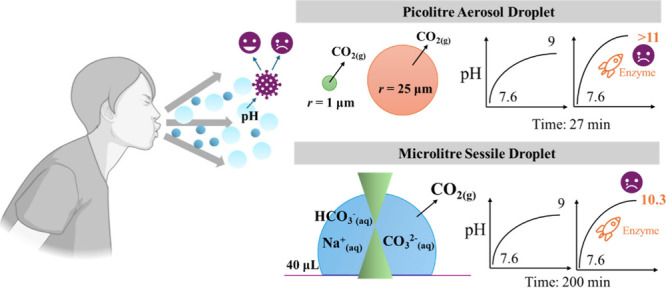

## 1 Introduction

Exhaled aerosols containing infectious pathogens play an important role in the transmission of respiratory diseases such as COVID-19.^1–3^ The aerosol microenvironment plays a central role in determining the longevity of airborne pathogens, depending, for example, on the composition of the aerosol, the relative humidity, temperature, and phase.^4–10^ Recent studies have discussed the role that the pH of exhaled aerosol plays in the loss of viral infectivity while airborne; indeed, aerosol pH is suggested as a significant factor in reducing SARS-CoV-2 as well as influenza A virus infectivity and aerostability.^6,11–13^ Sodium bicarbonate (NaHCO_3_) acts as a principal buffer in a range of biological systems, including in blood pH regulation and in the respiratory system, as well as in cell culture media, with the pH varying according to the amount of dissolved CO_2_, which in turn depends on the amount of gas phase CO_2_.^14–16^ In respiratory fluids, the sodium bicarbonate–carbonic acid (H_2_CO_3_) system is considered the primary buffer, more important than phosphate and proteins.^17–19^ Thus, understanding the buffering capacity of NaHCO_3_ and the response of respiratory aerosol pH upon exhalation into ambient environments is crucial to understanding viral survival in the aerosol phase.

Upon exhalation, the change in gas phase environmental conditions leads to a change in the exhaled droplet size, phase, and composition, including pH. In the human lung, the CO_2(g)_ level is about 50000 ppmV (5% atm),^20^ while in ambient air, the concentration of CO_2(g)_ ranges typically from 400 ppmV (0.04% atm)^21^ to several thousand ppmV^22^ in a crowded, poorly ventilated space. A high CO_2(g)_ concentration in the lung is in equilibrium with dissolved CO_2_ present as NaHCO_3_ according to Henry’s law, with 22–32 mmol/L being a normal bicarbonate level in adult blood.^23^ This corresponds to a physiological pH level around 7.1–7.3 given a Henry’s law constant of CO_2_ of 3 × 10^–2^ mol L^–1^·atm at 37 °C in lung fluid.^24^ The pH neutrality (6.7–7.3) of human saliva is maintained principally by bicarbonate in the oral cavity, and it has been suggested that the bicarbonate concentration is a function of saliva flow rate: the higher the flow rate, the higher the bicarbonate concentration.^21,25–28^ Bicarbonate buffer is also widely used in the incubation of cells in culture media, such as Dulbecco’s modified Eagle medium and minimal essential media (MEM) with a 5% atm or 2% atm CO_2(g)_ environment,^14^ to maintain the media pH through sustained equilibrium with gas phase CO_2_ for better cultivation results. These media are commonly used in aerosol viral infectivity studies.^6,8^

The equilibria leading to bicarbonate buffering are shown in eqs 1 and 2:

1 $${\mathrm{CO}}_{2\left(g\right)}+{H_{2}O}_{\left(l\right)}⇌H_{2}{\mathrm{CO}}_{3\left(\mathrm{aq}\right)}$$

2 $${2\mathrm{HCO}}_{3\left(\mathrm{aq}\right)}^{-}⇌{\mathrm{CO}}_{3\left(\mathrm{aq}\right)}^{2-}+H_{2}{\mathrm{CO}}_{3\left(\mathrm{aq}\right)}⇌{\mathrm{CO}}_{3\left(\mathrm{aq}\right)}^{2-}+{\mathrm{CO}}_{2\left(g\right)}+{H_{2}O}_{\left(l\right)}$$

The hydration of CO_2_ is an extremely slow process (eq 1),^29,30^ whereas the dissociation of H_2_CO_3_ (to HCO_3_^–^ and H^+^) is immediate (eq 2). In reverse, the dehydration of H_2_CO_3_ is also very slow. For exhaled aerosol, the HCO_3_^–^ in the droplet will diminish through the kinetically limited evaporation of dissolved CO_2_ into the gas phase due to the lower vapor pressure of CO_2_ in the ambient environment when compared with the lung (eq 2). However, both the forward and backward reactions are catalyzed by the enzyme carbonic anhydrase (CA) in human saliva and other biological systems,^31^ which accelerates this reversible process by a factor of ∼10^7^.^29^ This essential enzyme ensures that metabolic processes proceed at a sufficient rate during inhalation and exhalation to maintain physiological pH (e.g., in plasma and lungs) at a stable level.

Raman spectroscopy has been used in laboratory studies to investigate the pH dependence of atmospheric aerosol transformations.^32,33^ For example, glycine was used as an *in situ* pH probe to infer HCl and HNO_3_ depletion in microdroplets at the range of pH 1 to 4 (droplet radius: ∼3.5 μm).^32^ Craig et al. used Raman vibrational modes of *v*_s_(SO_4_^2–^) and *v*_s_(HSO_4_^–^) combined with pH indicator paper to measure the size-resolved aerosol acidity from pH 0 to 4.5 (droplet radius: 0.27–9.9 μm).^33^ Craig et al. also coupled Raman microspectroscopy with extended Debye–Hückel activity calculations to directly determine the acidity of individual particles for a wider range of pH 1 to 10 (droplet radius: 0.5–7.5 μm).^34^ Raman spectroscopy has also been used to characterize the local pH near an electrode surface. For example, phosphate species were utilized as a pH indicator in the range of 0.68 to 13.7.^35^ However, Raman spectroscopy has not yet been applied to study the pH of respiratory fluids or compositionally relevant surrogates.

The aim of this paper is to explore the HCO_3_^–^/CO_3_^2–^ equilibrium in surrogate respiratory fluid sessile droplets to provide information about the pH evolution of exhaled respiratory aerosol, yielding crucial insights into the aerosol processes that are central to airborne disease transmission. *In situ* direct measurements are made using Raman spectroscopy to study the kinetics of CO_2_ evaporation from NaCl-NaHCO_3_ microliter sessile droplets, with and without the enzyme CA. Specifically, we explore the impact of gas flow rate, droplet volume, gas phase CO_2_ concentration, and enzymatic catalyst concentration on CO_2_ evaporation kinetics. In addition, the role of phosphate buffer is examined. Detailed experimental methods and model simulations are described in the Supporting Information. Experimental pH measurements were compared with predictions from the ACCENT model—the European Network of Excellence—a Pitzer activity coefficient model used to estimate ion concentrations, activity coefficients, and equilibrium pH in NaCl-NaHCO_3_ mixing solutions. These measurements provide a robust foundation for benchmarking a fully Lagrangian kinetic model, the Respiratory Aerosol Model (ResAM), which simulates the thermodynamics and evolving acidity of respiratory aerosols. The uniqueness of this method lies in the direct use of the Raman signal from the solution, rather than relying on external pH indicators (e.g., gold nanoparticles in surface enhanced Raman spectroscopy studies^36^). This eliminates potential interference from the additional components. Lastly, the benchmarked aerosol model is used to predict the evolving pH of respiratory aerosol particles of picoliter volumes.

## 2 Results and Discussion

### 2.1 Calibration of Raman Measurement of NaHCO_3_ and Na_2_CO_3_ Concentrations

Previous work has identified distinctive spectroscopic bands for aqueous bicarbonate (HCO_3_^–^) and carbonate (CO_3_^2–^) ions in Raman spectra corresponding to different vibrational modes. Table 1 summarizes prior work that reports Raman shifts and their assignments for aqueous sodium bicarbonate (NaHCO_3_), sodium carbonate (Na_2_CO_3_), and water (H_2_O).^37–42^ The C–O^–^ symmetric stretch is most pronounced at ∼1060 cm^–1^ for the carbonate anion, and a sharp peak from excitation of the C–OH stretch is observed at ∼1010 cm^–1^ for the bicarbonate anion. As they are dissolved in water, a band from the O–H bending vibration of water is observed near 1640 cm^–1^. One consequence of a sample open to air is that a small amount of NaHCO_3_ will disproportionate to form CO_2_ and carbonate due to the much lower equilibrium vapor pressure of CO_2_ in the gas phase (eq 2); it is expected that the spectrum of a NaHCO_3_ solution will also contain a small peak attributable to carbonate.^38,43^ Between 1300 and 1500 cm^–1^ Raman shift, several bands overlap in solutions of both anions, assigned to bicarbonate vibrational modes that include C–OH bending, C=O bending, and the CO–H stretch.

**Table 1:** Spectroscopic Characteristics Values of Aqueous NaHCO_3_, Na_2_CO_3_, and H_2_O

Raman shift (cm^**–**1^)	1000–1200	1300–1500	1600–1700
NaHCO_3_	**C–OH stretch**	**C–OH bend**	
	1015 ^38,39^	1300 ^38,39^	
	1016 ^40^	1302 ^38^	
	1017 ^38^	**C**=**O bend**	
	1043 ^41^	1302 ^38^	
		**CO–H stretch**	
		1360 ^38^	
		1364 ^40^	
		**C**=**O symmetric stretch**	
		1360 ^39^	
Na_2_CO_3_	**C–O** ^**‑**^ **symmetric stretch**	**C–O** ^**‑**^ **antisymmetric stretch**	
	1060 ^44^	1365 ^42^	
	1063 ^42^	1385 ^40^	
	1066 ^40^	1410 ^42^	
	1067 ^38,45^		
H_2_O			**O–H bend**
			1640 ^37,39^
			1641 ^40^

A series of Raman spectra were acquired using varying concentrations of aqueous solutions of pure NaHCO_3_ and Na_2_CO_3_ to examine the relationships between solution concentration, pH, and Raman signal response (Figure 1). Figure 1a and b shows the concentration-dependent Raman spectra of NaHCO_3_ and Na_2_CO_3_ solutions, respectively. Consistent with Table 1, four prominent peaks are observed in the Raman spectra of NaHCO_3(aq)_, located at 1010, 1060, 1370, and 1640 cm^–1^. These four peaks correspond to the distinctive C–OH stretch at 1010 cm^–1^, a C–O^–^ symmetric stretch at 1060 cm^–1^ attributed to a small amount of CO_3_^2–^ formed by ion dissociation, a CO–H stretch at 1370 cm^–1^, and the water bending vibration at 1640 cm^–1^. For Na_2_CO_3(aq)_ (Figure 1b), the pronounced C–O^–^ symmetric stretch appears at 1060 cm^–1^, with a C–O^–^ antisymmetric stretch at 1370 cm^–1^, and the water bending vibration at 1640 cm^–1^. The Raman signal intensity depends on ion concentration, laser beam intensity, laser path length, the collection efficiency of the optical system, and the Raman cross-section of the species. In our measurements, the first four parameters are held constant, so it is evident that the Raman cross-section of CO_3_^2–^ is much higher than that of HCO_3_^–^. This is further illustrated in Figure 1c, which shows a linear relationship between the ratio of the carbonate or bicarbonate intensity to the water peak intensity and the solute concentration. The ratio of the slopes for the CO_3_^2–^ and HCO_3_^–^ Raman responses is ∼4.8, reflecting this difference in Raman cross-section. This has been explored in the crystalline phase as a function of pressure by Pan and Galli, who reported a cross-section ratio of ∼1.8 between the two ions.^46^ The corresponding bulk solution pH values measured at different concentrations are shown in Figure S3. The relationship between solution pH and concentration exhibits opposite trends for NaHCO_3_ and Na_2_CO_3_: the pH increases with Na_2_CO_3_ concentration but decreases with increasing NaHCO_3_ concentration. In combination with ACCENT model calculations, it is also confirmed that higher bicarbonate concentrations require extremely high CO_2_ vapor pressure to be sustained under equilibrium conditions (e.g., 370000 ppmV for a pH 7.6 HCO_3_^–^ solution).

**Figure 1:**
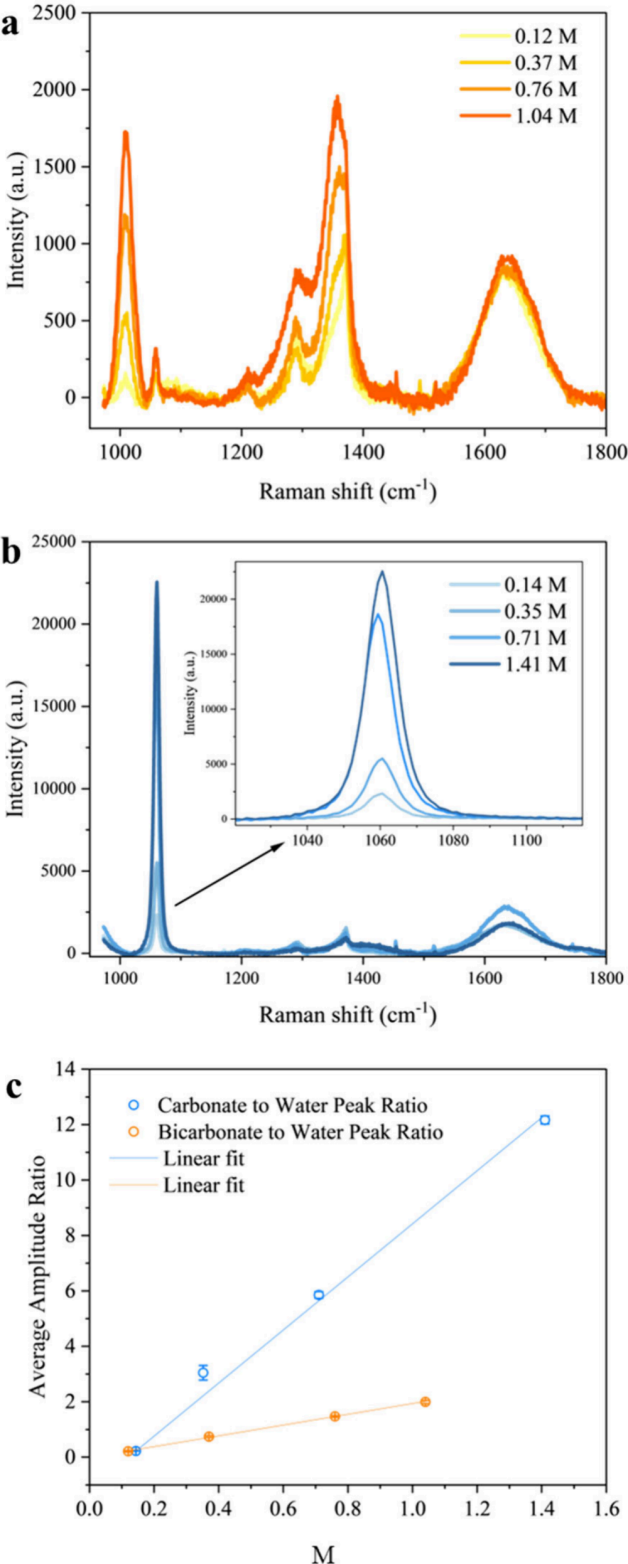
Concentration-dependent Raman spectra of (a) NaHCO_3_ and (b) Na_2_CO_3_ solutions with the corresponding Raman peak assignments are marked. The concentration unit is molarity (M). (c) Raman amplitude of HCO_3_^–^ (orange) and CO_3_^2–^ (blue) relative to the water peak (at 1640 cm^–1^) as a function of molarity. Each data point is an average of 3–5 measurements.

A second calibration procedure was performed for mixed solutions containing sodium chloride (NaCl), NaHCO_3_, and Na_2_CO_3_. Ten solutions were prepared based on equilibrium compositions that result from CO_2_ evaporates from the solution, i.e., at different HCO_3_^–^ and CO_3_^2–^ ratios. As CO_2_ evaporates, the concentration of Cl^–^ remains constant, while HCO_3_^–^ decreases and CO_3_^2–^ forms in the droplet. Figure 2 shows how the thermodynamic equilibrium composition of a NaHCO_3_-Na_2_CO_3_ solution varies along with the pH estimated from the ACCENT model calculation, which is compared to experimental measurements. The model input for solution composition of NaHCO_3_ is 0.51 mol kg^–1^ HCO_3_^–^ and then decreases to 0, while that of CO_3_^2–^ increases from 0 to 0.26 mol kg^–1^. The concentration of Cl^–^ remains at 1.47 mol kg^–1^. These values were chosen to match the composition of the solution that were prepared for Figure 2. From the ACCENT calculation, we can obtain the relative abundance of HCO_3_^–^ and CO_3_^2–^ in these solutions at equilibrium (Figure 2a). The relative abundance of a species is calculated by dividing its concentration by the total concentration of ions. When pH > 9.25, the HCO_3_^–^/CO_3_^2–^ equilibrium is increasingly on the carbonate side, and the ratio of [HCO_3_^–^] to [CO_3_^2–^] decreases exponentially with an increase in pH. On a logarithmic scale, the relationship is linear with solution pH (Figure 2b). The ACCENT estimated solution pH and the measured solution pH of the ten solutions are highly comparable, with the gradient of experimental and theoretical data close to 1 (*y* = *x*). These calibration measurements and calculations provide an important background for accurate interpretation of later results.

**Figure 2:**
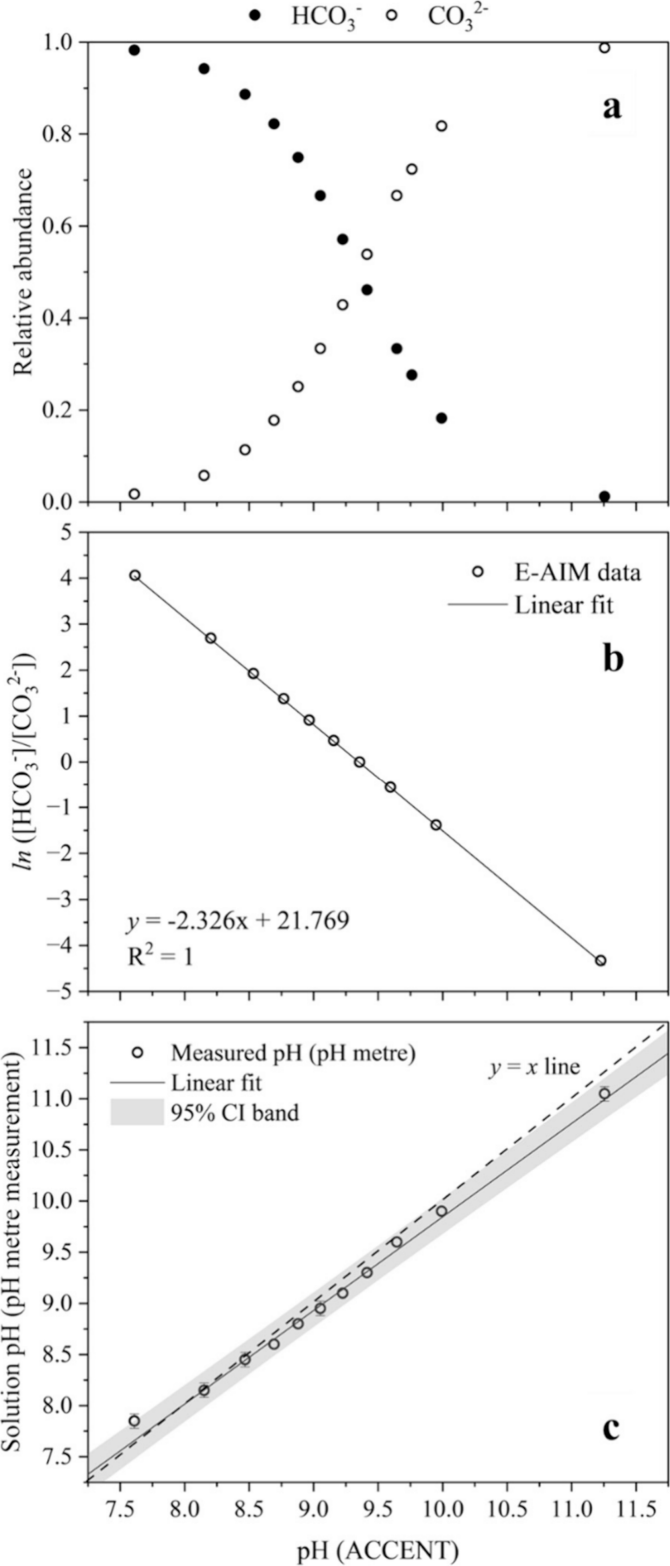
Thermodynamic equilibrium ACCENT calculation of the NaCl-NaHCO_3_ solution with varying [H^+^] and [CO_3_^2–^] content as CO_2_ evaporates until there is no HCO_3_^–^ left. (a) The relative abundance of HCO_3_^2–^ and CO_3_^2–^ ions at given pH conditions; (b) the logarithmic ratio of [HCO_3_^–^] and [CO_3_^2–^] at given pH values; (c) the ACCENT calculated equilibrium droplet pH compared to the bulk solution pH measurement using the pH meter.

### 2.2 Measurements of the Time Dependence of Sessile Droplet pH with no CO_2_ in the Gas Phase

#### 2.2.1 pH-Dependent Raman Spectra and Peak Intensity Ratio

To explore the CO_2_ evaporation kinetics from an aqueous NaCl-NaHCO_3_ droplet, a direct *in situ* approach for inferring the HCO_3_^–^ and CO_3_^2–^ ion concentrations and pH values must be established. Figure 3 reports the pH-dependent Raman spectra and the HCO_3_^–^-CO_3_^2–^ peak amplitude ratio for sessile droplets of solutions with the same compositions as those presented in Figure 2. The trend of the peaks in Figure 3a indicates that the HCO_3_^–^ peak intensity decreases as the pH increases, and the carbonate peak intensity becomes stronger, consistent with the droplets becoming more alkaline. When the droplet is strongly alkaline and entirely CO_3_^2–^ in composition, no HCO_3_^–^ peak is observed; the quantification of the HCO_3_^–^ peak intensity (and, thus, pH) is increasingly uncertain as the pH increases. The variation in the log of the intensity ratio is reported in Figure 3b and the point at pH 11.1 is excluded due to the uncertainty of the measurement. Figure 3b confirms that a linear relationship exists between the logarithm of the HCO_3_^–^-CO_3_^2–^ peak amplitude ratio and the droplet pH. Thus, the pH can be estimated for any given time point during the CO_2_ evaporation from an aqueous sessile NaCl-NaHCO_3_ droplet by using the HCO_3_^–^-CO_3_^2–^ peak amplitude ratio.

**Figure 3:**
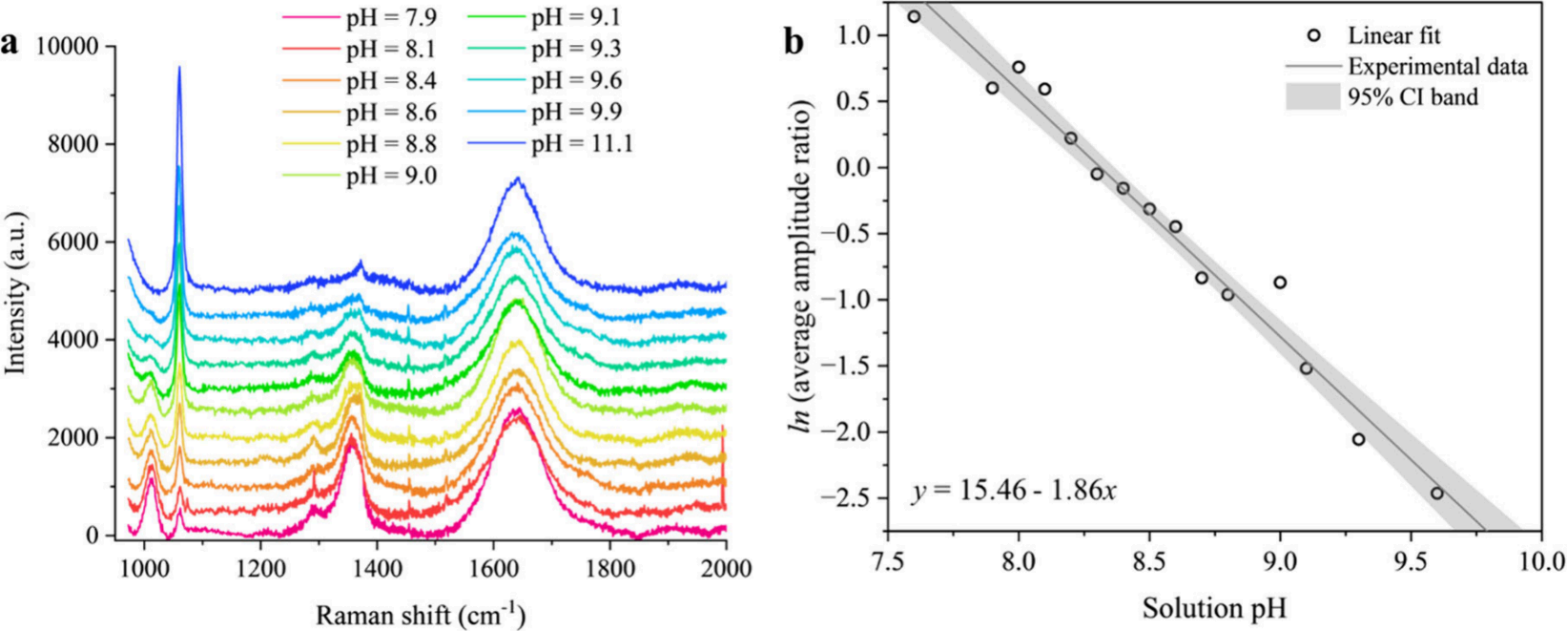
(a) The pH-dependent Raman spectra of the NaCl-NaHCO_3_ solution; (b) the peak amplitude ratio of HCO_3_^–^ and CO_3_^2–^ peaks in logarithm space as a function of pH. Each data point is an average of 3–5 measurements.

As a first demonstration, the time dependence of the spectrum and how it is used to retrieve pH are shown in Figure 4. Multiple long-time measurements have been repeated, with an example of a 16-h time-dependent Raman measurement. The intensity of the HCO_3_^–^ peak decreases over time, while the intensity of the CO_3_^2–^ peak increases significantly. The peak near 1370 cm^–1^ has contributions from both HCO_3_^–^ and CO_3_^2–^ vibrational modes, but the HCO_3_^–^ contribution is dominant, leading to a net decrease over time. The water bending peak intensity at 1640 cm^–1^ remains largely stable, with a slight increase over time likely due to subtle overnight environmental changes, such as a lower temperature and CO_2(g)_ evaporation, leading to a modest rise in the relative water concentration. By acquiring the peak amplitude ratios from the first two peaks (HCO_3_^–^-CO_3_^2–^), the pH change can be estimated, as shown in Figure 4b, using the relationship reported in Figure 3b. Figure 4b indicates that the droplet pH has increased to 10.4 from the initial pH of 7.95 after 16 h. It is also evident that the pH estimation is robust before ∼400 min, while the pH estimation becomes more scattered due to the challenge of accurately quantifying the small fraction of HCO_3_^–^ present after ∼400 min. The concentration and pH changes in the aqueous NaCl-NaHCO_3_ droplet are driven by the CO_2_ evaporation. The pH and Raman peak changes are similar to a study that investigated the local pH variation near the surface of a CO_2_ reduction electrode by Lu et al.^47^

**Figure 4:**
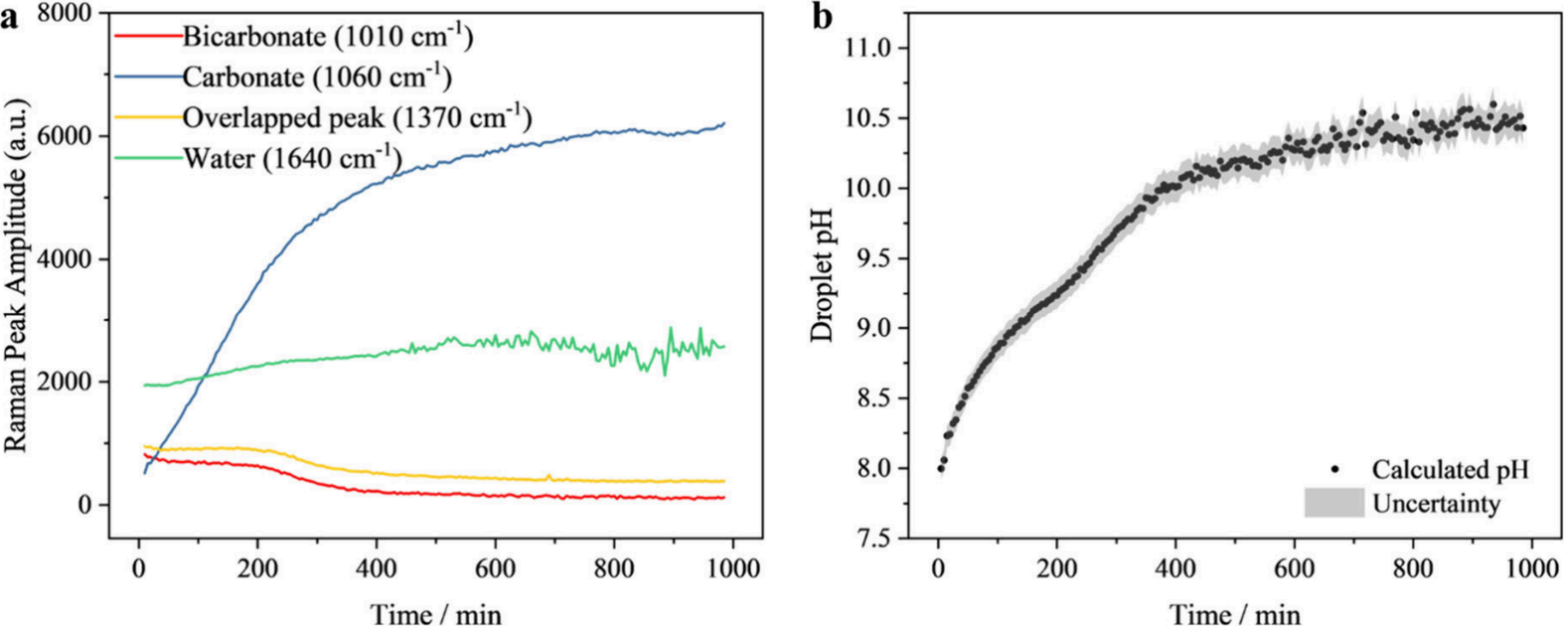
Quantifying the pH changes of the sessile droplet based on Raman peak amplitude measurements. (a) The changes in four Raman peaks (HCO_3_^–^ in red circles, CO_3_^2–^ in yellow circles, water in purple triangles, and overlapped peak in blue triangles) over the course of a 16-h long experiment; (b) the estimated pH change over time based on the measured Raman peak ratio for a NaCl-NaHCO_3_ droplet. The gray shaded area represents the uncertainty in the retrieved pH based on the calibration experiments, derived from the upper and lower bounds of the 95% CI of the linear fit shown in Figure 3b.

#### 2.2.2 Dependence of Compositional Change Kinetics on Gas Flow Rate and Droplet Volume

To test the robustness of this pH estimation method, the sensitivities to the gas flow rate and droplet volume are explored (Figure 5). For the gas flow rate, constant flow rates at 0, 50, 100, and 200 sccm are investigated (Figure 5a). The initial compositions of the droplet are 1.47 mol kg^–1^ NaCl and 0.51 mol kg^–1^ NaHCO_3_, with a pH of ∼7.6. The results indicate that there is no systematic change in the variation in pH with time with varying gas flow rate. In fact, the variability in the peak amplitude ratio and pH change at different gas flow rates is within the range of experimental error, estimated from the standard deviation (SD) of six measurements at a 200 sccm gas flow rate (Figure 5a, gray shaded area). The individual measurements are shown in Figure S4. This suggests that the proposed approach for estimating the droplet pH is independent of the gas flow rate passing over the droplet.

**Figure 5:**
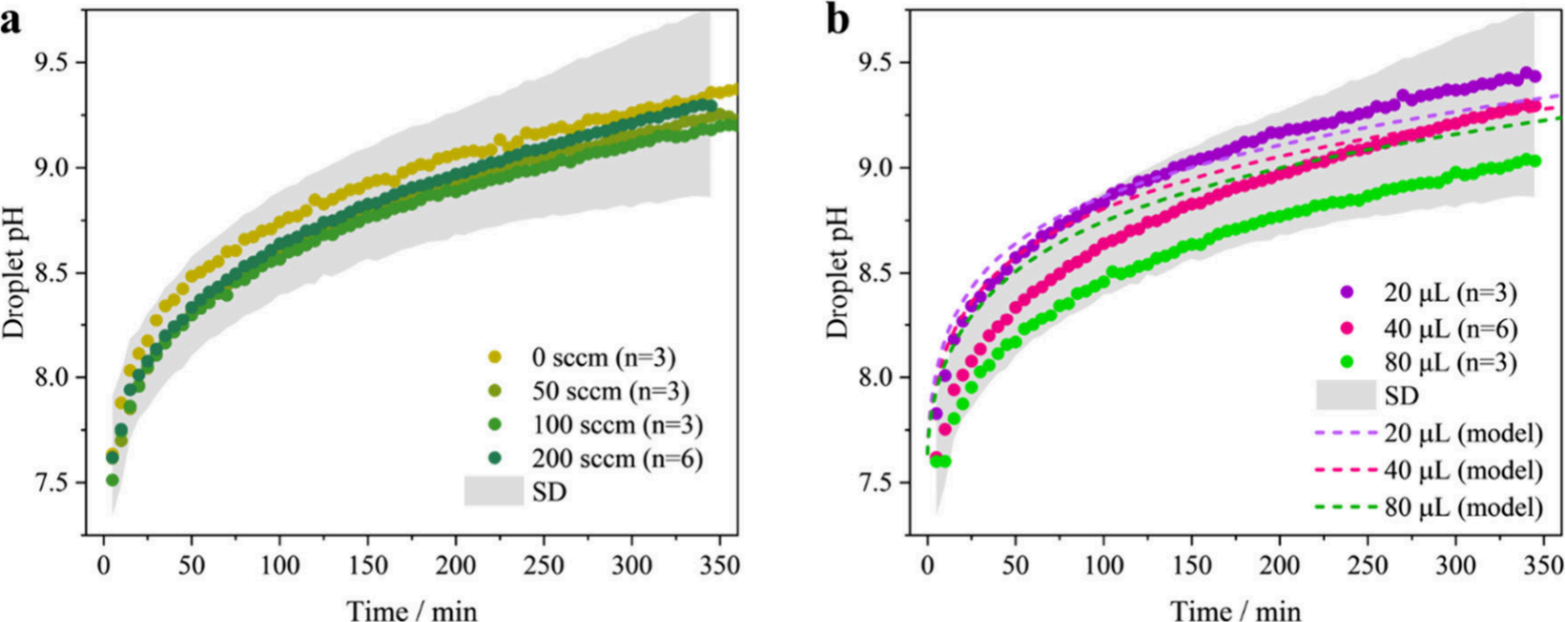
Sensitivity to gas flow rate and droplet volume: (a) droplet pH over time at varying gas flow rates, including 0, 50, 100, and 200 sccm; (b) the pH of sessile droplets with volumes of 20 (purple), 40 (pink), and 80 (green) μL (circles, experimental data) and compared with modeled data (dashed lines). The gas flow rate for experiments in (b) are all at 200 sccm.

Regarding the measured time dependence of pH with droplet volume, we observed that larger droplets exhibit slower pH increases (Figure 5b, circles). This trend is qualitatively captured by the ResAM model (Figure 5b, dashed lines), which predicts a modest difference in the pH evolution for μL-scale droplets. However, statistical analysis using the Mann–Whitney U test indicated that these differences are not statistically significant (*p* > 0.05) and may fall within the measurement uncertainty. Upon close inspection, the model shows a higher pH increase rate in the first 20 min. The discrepancy may be attributed to uncertainty in the experimental in *t* = 0 s; the first Raman data point may occur slightly after 5 min due to the time required for experimental setup and alignment.

### 2.3 CO_2_ Evaporation Kinetics of Sessile Droplet with Changing CO_2(g)_ Concentration

The experiment was then conducted in a CO_2_-controlled environment to examine the impact of gas-phase CO_2_ concentration on the pH increase in droplets with the same initial composition as the previous measurement, and to compare the results with model predictions (Figure 6). For a given aqueous NaCl-NaHCO_3_ droplet, the equilibrium vapor pressure of CO_2_ (*p*_0_^CO_2_^) in the gas phase can be extremely high, up to 370000 ppmV, when the droplet pH is 7.6. In the humid lung, *p* ^CO_2_^ can reach up to 50000 ppmV,^21^ which leads to equilibration with dissolved CO_2_ and the HCO_3_^–^-CO_3_^2–^ buffer, resulting in a pH of 8.5 (ACCENT model). In the laboratory, two significantly different CO_2(g)_ levels were investigated: measurements were made for a 40 μL NaCl-NaHCO_3_ droplet equilibrating with a gas flow rate of 200 sccm with either 5000 or 25000 ppmV of CO_2_. According to ACCENT model calculations, the droplet compositions should equilibrate at pH 8.7 and 9.1 at these two CO_2(g)_ levels. Measurements indicate that the droplet pH increased to 8.6 and 9.0, respectively, at 420 min, values that closely match the ACCENT model predictions. This result supports the influence of the gas-phase CO_2_ concentration on both the rate of pH increase and the final equilibrium pH of the droplet. According to Le Châtelier’s Principle, a higher CO_2(g)_ concentration suppresses CO_2_ release from the droplet, thereby stabilizing its composition and pH.

**Figure 6:**
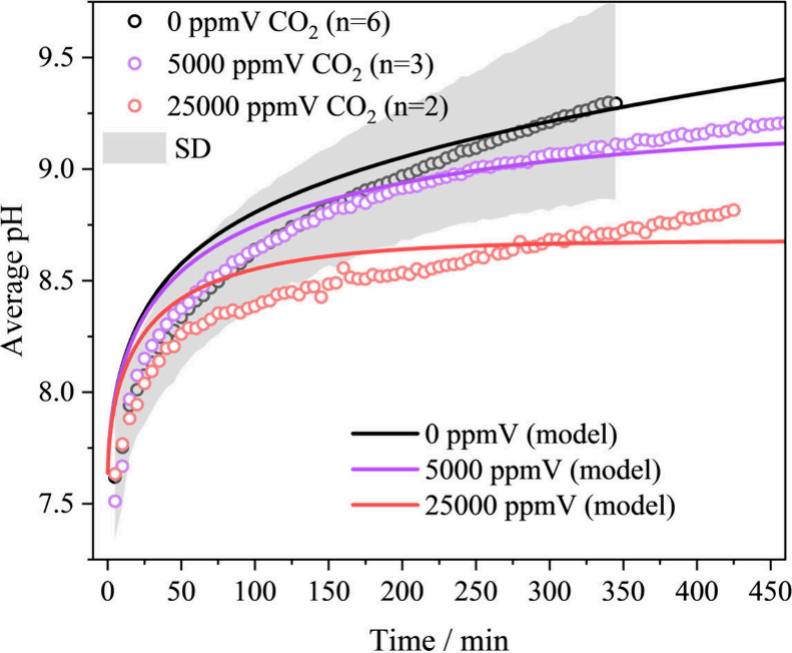
Measured (circles) and modeled (solid lines) changes in pH over time for sessile droplets containing 1.47 mol kg^–1^ NaCl and 0.51 mol kg^–1^ NaHCO_3_ under different CO_2_ gas conditions: 0 (black), 5000 (purple), and 25000 ppmV (red).

The CO_2_-dependent pH change of the aqueous sessile NaCl-NaHCO_3_ droplet is also modeled by the ResAM model. The simulation agrees with the trend of the experimental measurements, but a faster increase in pH in the first 20 min is still observed. However, the measured and modeled equilibrium pH values approach very similar values after 300 min at 0 and 5000 ppmV, with the model predicting equilibrium being reached earlier than observed in the experiment at 25000 ppmV.

### 2.4 Fast CO_2_ Evaporation Kinetics with Carbonic Anhydrase (CA) Enzyme Presence

CA exists in various biological systems, such as human saliva, and acts as a catalyst for CO_2_ gas hydration or H_2_CO_3_ dehydration reaction.^48^ Thus, the effect of CA on the CO_2_ evaporation rate and pH change at the two CO_2_ levels is explored. One set of experiments was conducted at 400 ppmV of CO_2_, while the rest were conducted at 0 ppmV (Figure 7). Results show that adding trace amounts of CA solution (0.015% *w*/*w* and 0.077% *w*/*w*) can significantly accelerate the CO_2_ evaporation kinetics significantly. Here, we use ‘fast kinetics’ to refer to measurements with added CA, and ‘slow kinetics’ to refer to measurements without added CA. We also use ‘high CA’ to refer to the 0.077% *w*/*w* CA solution, ‘low CA’ to refer to 0.015% *w*/*w* CA solution, and ‘no CA’ to refer to the slow kinetics measurement. The CO_2_ evaporation fast kinetics are very reproducible (Figure S5a), achieving pH 9 after only 50 min, which is 5 times faster than the slow kinetics. The fast kinetic measurements indicate that the upper limit of the pH can be controlled by both the enzyme concentration and the HCO_3_^–^ concentration in the droplet. It has been reported that the optimal pH for CA performance is 8.1.^49,50^ In this experiment, the higher enzyme concentration results in the pH approaching and exceeding 11, which is higher than the pH observed in the long term slow kinetic measurement (Figure S5b). The rate of pH increase decreases over time, attributable to the depletion of CO_3_^2–^/HCO_3_^–^ as the CO_2_ evaporates off.

**Figure 7:**
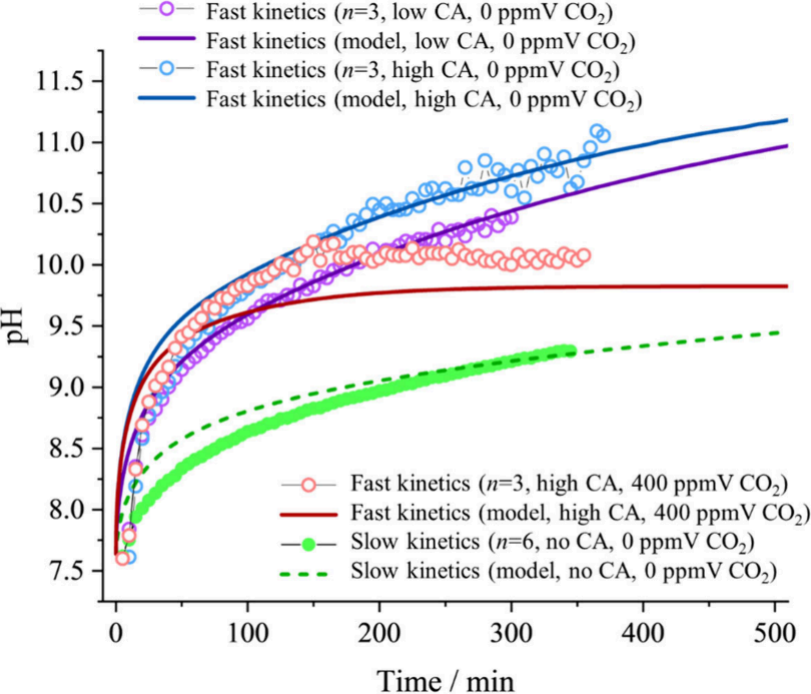
Measured (circles) and modeled (solid lines) changes in pH with time for solutions with bicarbonate buffer only (green), bicarbonate buffer with 0.015% *w*/*w*) CA (purple), bicarbonate buffer with 0.077% *w*/*w* CA (blue), and bicarbonate buffer with 0.077% *w*/*w* (high) CA surrounded by 400 ppmV CO_2_ (red).

At a 400 ppmV CO_2_ level (ambient CO_2_) and with 0.077% *w*/*w* CA added, the final droplet pH is about 1 unit lower than when there is no CO_2_ in the gas phase: the pH increases to 10.26, whereas measurements in the absence of CO_2_ rise to 11.05. Additionally, the initial rate of pH increase for the droplets containing 0.077% *w*/*w* CA is similar at both 0 and 400 ppmV gas phase CO_2_ concentration. This indicates that the gas phase CO_2_ level does not affect the reaction rate but has a notable impact on the final pH of the droplet.

Assuming a molar mass of 3 × 10^5^ g mol^–1^ for the CA enzyme, the concentration of [*E*] in eq S5 can be readily calculated (see figure caption in Figure 7). A catalytic rate $\frac{k_{cat}}{K_{m}}$ of 10^5.45+0.1×(pH‑7)^ s^–1^ kg mol^–1^ can be obtained. The simulation results using the rate coefficient reported by Khalifah et al.^29^ for HCA-B ( $\frac{k_{cat}}{K_{m}}$ = 10^6.7+0.4×(pH‑7)^ s^–1^ kg mol^–1^) are shown in Figure S9, which is a factor of 35 higher at pH 8 and 160 higher at pH 10 than the results of present study. The rate coefficient is so high that it becomes liquid phase diffusion limited, resulting in practically no difference between high and low CA concentrations. Conversely, faster liquid phase diffusion leads to an increasing difference between low and high CA concentrations (Figure S7). The data presented here constrain both the liquid phase diffusion and the catalytic rate coefficient. The difference between the two simulated curves with CA enzymes at 0 ppmV CO_2_ (blue and purple) becomes larger than the measurement. In Figure 7, with a reduced diffusion coefficient for ions, the system is partly liquid phase diffusion limited and agrees with the measured data.

These experimental results are further compared to the ResAM simulations (Figure 7). The results indicate that the fast kinetic data from experiments and simulations are in strong agreement when there is no gas phase CO_2_ for both CA concentrations. However, when the gas phase CO_2_ is at 400 ppmV, a discrepancy arises after about 60 min. During the first 60 min, the simulation closely matches the experimental data, but thereafter, the simulation equilibrates toward 9.8, while the experimental data reaches approximately 10.07 (±0.11). Michaelis–Menten kinetics was used to describe enzyme-catalyzed reactions involving one substrate and one product.^51^ For the CO_2_ hydration process (eq 1), the substrate is CO_2(g)_ and the enzyme is CA, which forms the final product H_2_CO_3_. Given the very low enzyme concentration, the enzyme-catalytic reaction rate should vary linearly with the substrate concentration. When considering the experimental measurement uncertainty (±0.11 pH unit), the model still cannot fully explain the discrepancy observed in the fast kinetics results at 400 ppmV gas phase CO_2_, leaving a discrepancy of approximately 0.15 pH unit. It is important to note that the fast kinetics of H_2_CO_3_ dehydration by CA could be even faster as multiple types of CA are present in human respiratory fluid, each with different rate constants. Consequently, the final pH in real respiratory aerosols may rise more quickly and reach even higher levels.

Additionally, we investigated the impact of an added phosphate buffer. In the artificial saliva formulation by Woo et al.,^52^ phosphate buffer is present alongside a bicarbonate buffer, with concentrations of 0.42 g/L NaHCO_3_, 0.21 g/L KH_2_PO_4_, and 0.43 g/L K_2_HPO_4_. Similar CO_2_ evaporation kinetics were observed, as shown in Figure 8. As with the bicarbonate buffer alone, the HCO_3_^–^ Raman peak decreases over time, while the CO_3_^2–^ peak increases (Figure 8a). However, the phosphate buffer does not affect the pH increase of the droplet, with changes closely resembling those observed in the bicarbonate-only case (Figure 8b). This is consistent with previous findings that, although bicarbonate, phosphate, and proteins are the three major buffers in human saliva, the bicarbonate buffer plays the dominant role and contributes the greatest buffer capacity, governing pH changes in exhaled droplets.^21^

**Figure 8:**
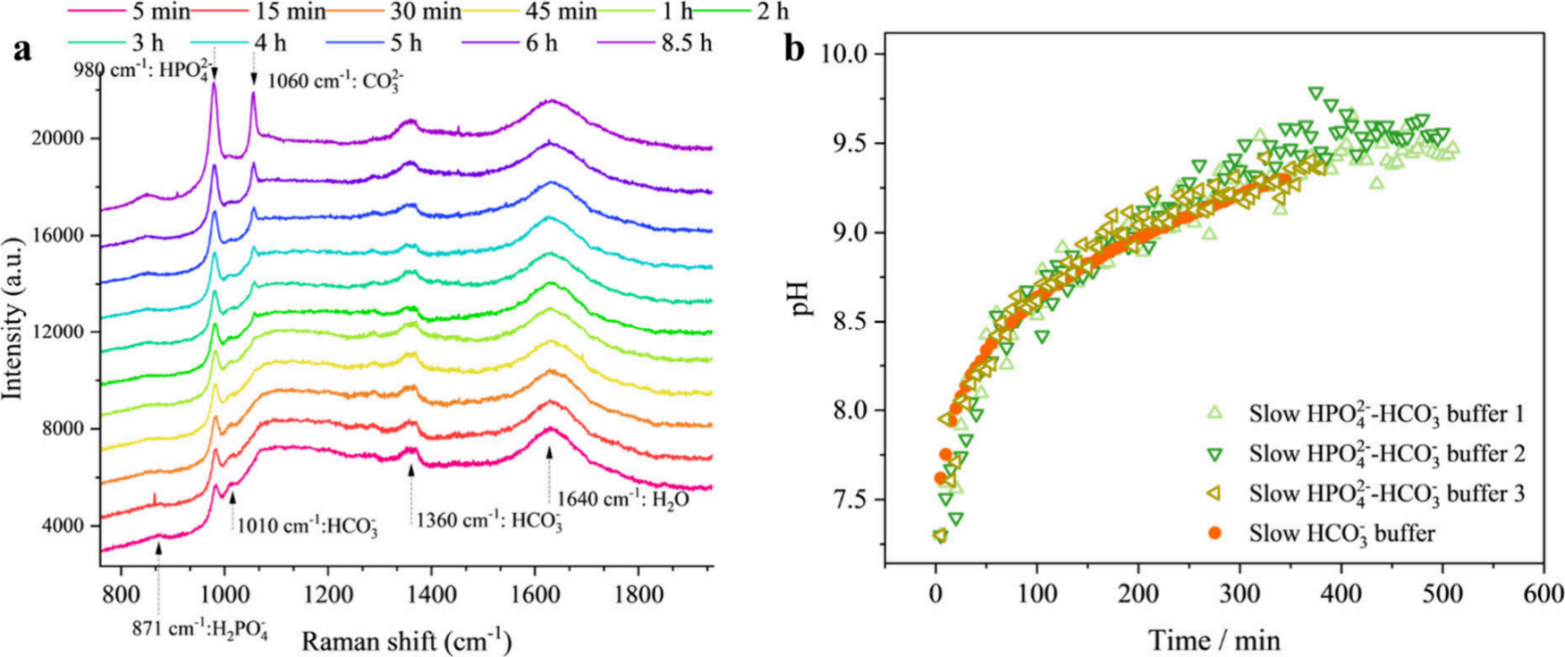
(a) Time-dependent Raman spectra of the phosphate-bicarbonate buffer. The spectra, displayed from bottom to top with line colors varying from pink to yellow-green and violet, corresponding to time points from 5 min to 8.5 h; (b) measured pH changes over time, comparing the phosphate buffer (light green-, green-, and olive-colored triangles, open symbols) with the pure bicarbonate buffer (solid bright orange circles).

### 2.5 pH Evolution Profile of Picoliter Aerosol Droplets

Having benchmarked ResAM, we now model the pH evolution profile for picoliter droplets with radii of 1 and 25 μm under both 0 and 400 ppmV CO_2_ conditions. These droplets are ∼10^5^ times smaller (25 μm radius) and ∼10^8^ smaller (1 μm radius) in volume than those used in the Raman measurements. Simulations are performed with and without a high CA concentration (0.077% *w*/*w*), using the same initial composition as in the Raman measurement (Figure 9). It is clear that, in the absence of CA, the pH increase is independent of droplet size. The droplet pH reaches 9 in ∼27 min in the aerosol phase, compared to ∼200 min in a microliter droplet with the same initial composition. When a high concentration of CA is present, the pH increases much more rapidly and reaches a higher final value. A droplet containing high CA reaches pH 9.8 in just 8 min. However, the actual pH may even be higher, as suggested by the discrepancy between the modeling results and laboratory measurements in Figure 7, where the measured pH of a droplet containing high CA at 400 ppmV reaches approximately 10.3, while the model predicted only 9.75.

**Figure 9:**
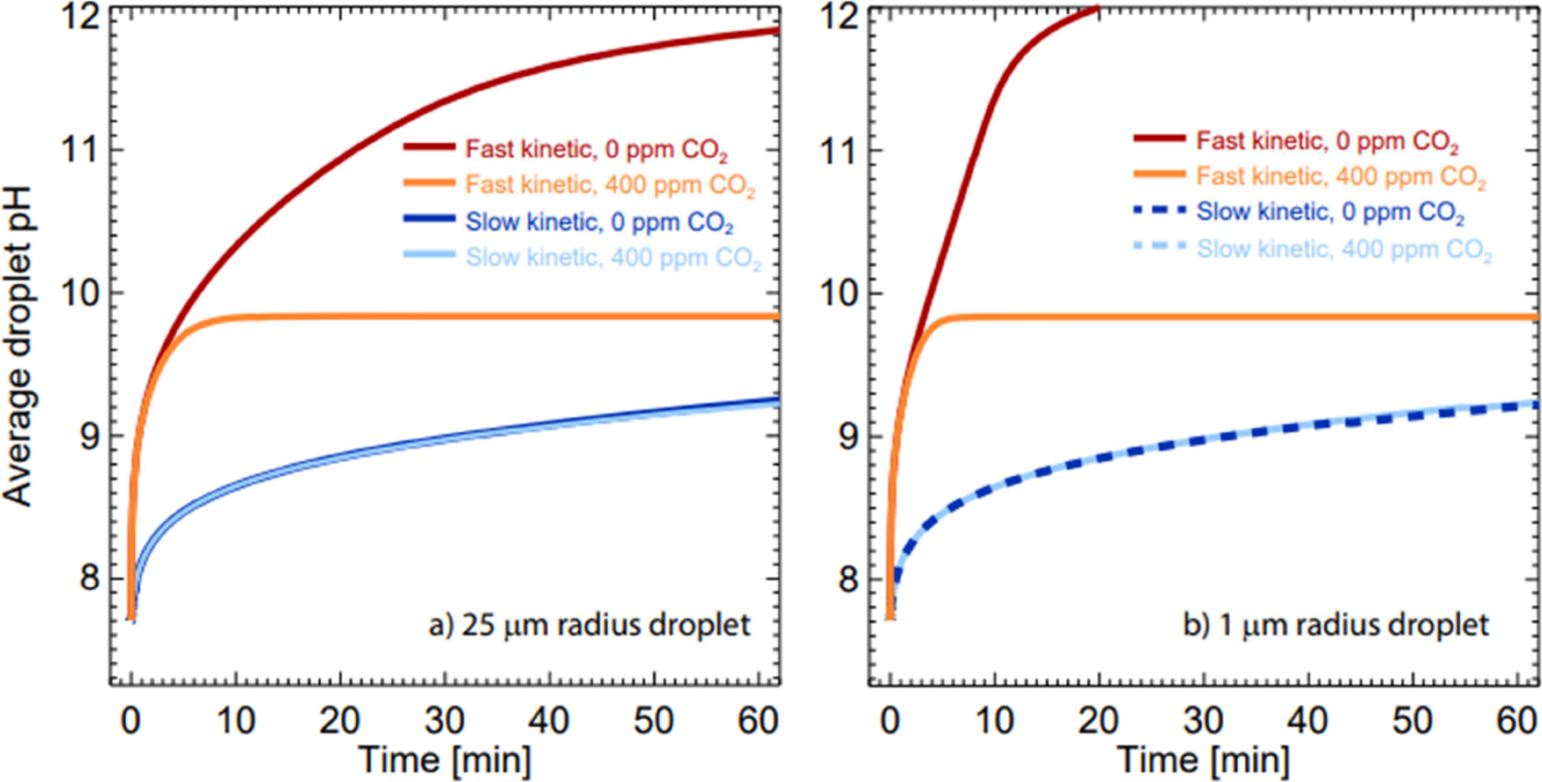
Simulated pH evolution profiles of microdroplet of (a) 25 μm radius and (b) 1 μm radius, at 0 and 400 ppmV CO_2_ conditions, with (in dark red and orange color) and without high CA (in light blue and blue color).

Complementary aerosol Raman measurements of pure Na_2_CO_3_ solution droplets, taken within 5–10 min after aerosolization and displayed in Figure S6, reveal that even when aerosol droplet and sessile droplets originate from solutions with the same solute concentration, the aerosol droplets exhibit a higher carbonate-to-water peak ratio almost immediately due to rapid water evaporation. Since the solution pH is inversely correlated with the logarithm of the HCO_3_^–^-CO_3_^2–^ peak amplitude ratio, this elevated carbonate-to-water signal in the aerosol phase is consistent with a higher pH. In exhaled respiratory aerosols, water activity (*a*_w_) decreases significantly as a result of evaporation. However, data on equilibrium pH and CO_2_ vapor pressure at low RH/*a*_w_ are scarce, as existing models are typically parametrized using measurements from high *a*_w_ solutions. Consequently, model predictions at low *a*_w_, typical for ambient aerosol, are often based on extrapolations rather than direct measurement, introducing non-negligible uncertainty.

The increased uncertainty at typical ambient RHs that respiratory aerosol is dispersed in is highlighted in Table S2, which provides a comparison between predictions from the ResAM, ACCENT and MarChemSpec (MCS) models at varying *a*_w_. The uncertainty of the prediction at low *a*_w_ is high (*a*_w_ < 0.7). For example, at same input conditions, the estimated pH can vary by up to 1.58 pH unit (data on the bottom row) with ResAM providing a much lower estimate of the pH than the models that include more recent thermodynamic measurement data. There are several potential sources of uncertainty: 1) NaHCO_3_ solubility is very low at low *a*_w_ (1 mol·kg^–1^ H_2_O); 2) the osmotic coefficients of NaHCO_3_ from 1 to 15 mol·kg^–1^ H_2_O was extrapolated; 3) in the present version of ResAM, the interaction parameters of CO_2_ and Na^+^ is from dos Santos et al.,^53^ that is based on Na^+^ concentration below 8 mol·kg^–1^ H_2_O. Because our experiments are conducted at a high *a*_w_ of 0.93, the models show good agreement. However, this limitation cannot be ignored, and it is important to recognize that these discrepancies present challenges in accurately predicting the pH and vapor pressure of aerosols relevant to atmospheric and respiratory processes. Additionally, we acknowledge there may be inherent errors in aerosol infectivity studies conducted at low RH or low *a*_w_. To address this, experimental validation of the model prediction at low RH is needed in future studies. Another limitation of this study is that we focused on a simplified but representative system, considering only ion interactions between Na^+^, Cl^–^, HCO_3_^–^, and CO_3_^2–^. In actual respiratory fluid, trace ions such as Ca^2+^ and Mg^2+^ may influence ion interactions—for example, by promoting the precipitation of solids (e.g., CaCO_3_)—which could in turn affect the pH evolution of exhaled aerosols. The formation of such solids may also negatively impact the viral survival, as the crystallization process can destabilize virions by altering local microenvironments or through physical entrapment.^6,54^

Additionally, the gas phase CO_2_ level significantly affects the final droplet pH. This finding agrees with the microliter sessile droplet measurements presented in the earlier results. These findings further demonstrate that CO_2_ evaporation is restricted by chemical kinetics rather than by a diffusion-limited reaction, where the enzyme CA largely accelerates the dehydration of H_2_CO_3_ to form the CO_2_ gas. As these reactions (hydration of CO_2_ and dehydration of H_2_CO_3_) do not contain a mass transport component and the forward and reverse rates are defined with respect to concentration, they should proceed at the same rate irrespective of droplet size. Since only CO_2_ can cross the liquid/gas boundary, mass transport only goes so far in increasing the kinetics of this reaction.

The time scale of pH increases within aerosol droplets containing bicarbonate buffer matches closely the infectivity decay profile from several studies that reported the aero-stability of SARS-CoV-2 viruses in the aerosol phase. For example, Haddrell et al.^11^ reported the infectivity of the SARS-CoV-2 original and Delta strains at 90% RH and suspended in MEM droplets. MEM is a type of cell culture medium that contains various inorganic salts with NaHCO_3_ as the major buffer for pH. A fast infectivity decay was observed in the first 10 min of measurement, followed by a moderate decay, then a very slow decay rate.

### 2.6 Significance of Understanding the Respiratory Aerosol pH Evolution Time Scale and Viral and Bacterial Viability

The improved understanding of the pH dynamics of exhaled respiratory aerosol droplets reported here has direct implications for how airborne microbe decay studies should be interpreted. First, the common assumption that acidity plays the sole role in driving aerosol pH under normal atmospheric conditions is incomplete.^55^ Although there exists an innate primary process driving respiratory aerosol toward alkaline pH, the environment must be at least somewhat polluted with condensable acidic vapors for the aerosol pH to become acidic.^12^ The degree to which alkalinity could explain the reported decay rates of exhaled viruses and bacteria is largely unexplored, with the few studies that have explicitly explored this area reporting a range of behaviors. For example, the loss of infectivity for SARS-CoV-2 can be attributed to the tendency to high pH of exhaled aerosol and the loss of infectivity of the virus at pHs above ∼9.5 to 10.^6^ Indeed, even subtle changes in CO_2_ concentration have been shown to have a profound impact on the decay dynamics by sustaining the bicarbonate pH buffer in the aerosol solution phase.^13^ Influenza has been reported to be sensitive to low pH (pH 4, with a ∼2-log_10_ reduction in viral titer after 30 s),^56^ but largely insensitive to neutral or high pH, while the high salt concentration in exhaled aerosol also plays a vital role in viral stability.^57^ Group A streptococcal (GAS) bacteria, unlike SARS-CoV-2 and influenza, have been shown to be highly insensitive to both high salt concentration and high pH,^58^ but a combination of high salt and high pH has been found to cause a dramatic loss in bacterial viability. These few studies demonstrate that the effect of high alkalinity on exhaled microbe decay is microbe dependent. Furthermore, as shown by the GAS study, while high pH may not be the primary driver of decay, it should still be considered when identifying the underlying decay mechanisms. Broadly speaking, the number of studies on microbial decay in solutions across the full pH range that could be accessed by exhaled aerosols is extremely limited, highlighting the need for more research into these relationships. It should also be recognized that the time scale for pH change is strongly coupled with time scales for chemical (e.g., water evaporation, acidic vapor condensation) and biological (e.g., structural changes in the integrity of a virion) change, leading to a complex interplay of processes that can only be more fully studied through aerosol phase measurements and precluding a complete analysis here.

## 3 Conclusions

This study presents the first direct *in situ* investigation of the HCO_3_^–^/CO_3_^2^^–^ equilibrium and CO_2_ evaporation kinetics in a NaCl-NaHCO_3_ surrogate respiratory fluid using Raman spectroscopy on sessile droplets. By analyzing the Raman peak amplitude ratio of HCO_3_^–^ to CO_3_^2–^, we tracked pH evolution under stable RH conditions, minimizing water loss. Measurement robustness was confirmed across varying gas flow rates and droplet volumes, with changes falling within experimental uncertainty. We assessed two key factors influencing droplet pH: ambient CO_2_ levels and the catalytic effect of CA, as well as the role of phosphate buffer. Results show that gas-phase CO_2_ strongly influences the final pH, while CA significantly accelerates the pH increase. Ultimately, the final pH depends on the CO_2_ levels, CA activity, and residual HCO_3_^–^ content.

The bicarbonate buffer capacity and enzymatic effect have only been studied in the field of dentistry, as saliva flow rate and calcium concentration affect the formation of calculus on teeth, which is important for oral health.^31,59^ The bicarbonate buffer, enzyme CA, CO_2_ evaporation, and acidity from exhaled droplets have been largely unexplored within the context of airborne disease transmission. Studies show that many viruses are pH sensitive and that high pH is a driving factor for the loss of infectivity for SARS-CoV-2 and its variants, and the ambient CO_2_ level affects the indoor airborne transmission infection rate.^11,13^

The composition of human respiratory fluid is dynamic, so reflecting the true nature and physicochemical properties of respiratory aerosols is challenging. For example, bicarbonate and CA contents are variable in human respiratory fluid, so it is possible that the infectious viruses in exhaled respiratory aerosols can be in different status (active or less active) when being generated, leading to different infectious rates. With changes in pH and the composition of real respiratory aerosols, it is possible that some other components, such as calcium and phosphorus, might precipitate, leading to more complex phase behavior of the droplet. Future studies are needed to address these questions. However, this study does highlight the time scale differences for the pH increase in microliter sessile droplets, which are more relevant to fomite transmission, and aerosol droplets of exhaled size, which are responsible for airborne transmission. It takes much longer for a microliter droplet (∼200 min) to reach the same pH level (e.g., pH 9) when compared to a picoliter aerosol droplet (27 min), indicating that some pH-sensitive viruses can potentially survive much longer in sessile droplets. This also highlights the need for care when interpreting pathogen survival times in microliter droplets deposited on surfaces and the extrapolation of these measurements to interpret survival in picoliter aerosols. There are numerous studies which may not fully account for the difference in time scale for pH increases when exploring the microphysical properties changing during drying,^60,61^ and also the survival of viruses in sessile droplets.^7,62^

This active and evolving field of research is marked by rapidly advancing methodologies and controversial views.^63,64^ Studies have shown that controlling indoor CO_2_ levels can reduce the risk of SARS-CoV-2 infection.^13^ This work bridges a critical gap in understanding the infection process, particularly the previously unknown time scale of pH changes. While high pH as well as low pH are known to inhibit viral survival in aerosols, key questions remain: How quickly does this change occur, and what factors influence it?

This study provides fundamental measurements of pH change time scales and CO_2_ partitioning between liquid and gas phases. It offers unique insights into exhaled respiratory aerosols, including bicarbonate buffering, CA effects, compositional changes, and pH evolution, connecting physiology, analytical science, and disease transmission. These measurements also lay a robust foundation for benchmarking the ResAM kinetic model of respiratory aerosol acidity and provide valuable data for biophysical and infectious risk models related to airborne disease transmission.

## Supplementary Material



## Notes

Experimental methods, including experimental details for Raman spectroscopy for sessile droplet measurements, chemical solutions, gas phase conditions; and model simulation details for ACCENT and ResAM models, and statistical test for Figure 5b (PDF)
