# Supplementary material for: Measurements of Surrogate Respiratory Sessile Droplet pH and Implications for Exhaled Respiratory Aerosol and Airborne Disease Transmission
Source: ACS Cent Sci. 2025 Jun 2;11(6):1009–19. doi: 10.1021/acscentsci.5c00284 (PMC12203433; doi:10.1021/acscentsci.5c00284)
Supplement: Supplementary file 1 [file oc5c00284_si_001.pdf]

Measurements of Surrogate Respiratory Sessile Droplet pH and Implications for Exhaled Respiratory Aerosol and Airborne Disease Transmission

Jianghan Tian[1], Beiping Luo[2], Aidan Rafferty[3], Allen E. Haddrell[1], Ulrich K. Krieger[2], Jonathan Reid[1,*]

[1]School of Chemistry, University of Bristol, Bristol, BS8 1TS, United Kingdom

[2]Institute for Atmospheric and Climate Science, ETH Zürich, CH-8092, Zürich, Switzerland

[3]Physical and Theoretical Chemistry Laboratory, South Parks Road, OX1 3QZ, United Kingdom

[*]Corresponding Author: Jonathan P. Reid (j.p.reid@bristol.ac.uk)

Supporting Information

S1. Experimental Methods

S1.1. Experimental Details

S1.1.1. Raman Spectroscopy of Sessile Droplets

The dependence of sessile droplet pH on bicarbonate concentration was measured with a commercial Aerosol Optical Tweezers coupled with Raman spectroscopy (Biral, AOT-100) by identifying vibrational signatures of different component ions in an aqueous solution of 2:1 mass ratio NaCl-$NaHCO_3$. This ratio is approximately equal to that of surrogate respiratory aerosol and cell culture media such as Dulbecco's Minimum Essential Medium (6.4 g L^{-1} sodium chloride, 3.7 g L^{-1} sodium bicarbonate). The AOT-100 has been applied to studies of phase separation[1] and coalescence[2] in the aerosol phase, but it also allows sessile droplet Raman spectra measurements to characterise the chemical composition of a given solution. For a typical experiment in this paper, a 40 μL droplet is placed on top of a Teflon sheet which is tightly attached to the coverslip in the trapping cell.

Raman spectra are taken in the backscattering geometry with a 532 nm Nd:YVO_4 laser (Opus 2W, Laser Quantum), propagating upwards in an inverted microscope through a coverslip and into an environmentally controlled cell. The laser is tightly focused through an air immersion objective (100× magnification, 0.95 NA), forming a focal point of high intensity within the sessile droplet. The laser power in all experiments was set to 50 mW, and the integration time for acquiring a Raman spectrum was set as 300 seconds to achieve a good signal-to-noise ratio. The backscattered Raman light is dispersed by a 1200 g/mm grating in a spectrograph and imaged onto a charge coupled device (CCD),

yielding a spectrum covering a wavelength range of 39 nm from, typically, 554 to 593 nm equivalent to a Raman shift of 746 to 1933 cm^{-1}. A Raman baseline subtraction was processed using custom scripts in MATLAB R2023b based on Mazet.[3] The estimation of the Raman peak amplitude was also performed using custom scripts, with the Raman peaks each fit to a Gaussian function. An example of the results of baseline subtraction is shown in Figure S1.

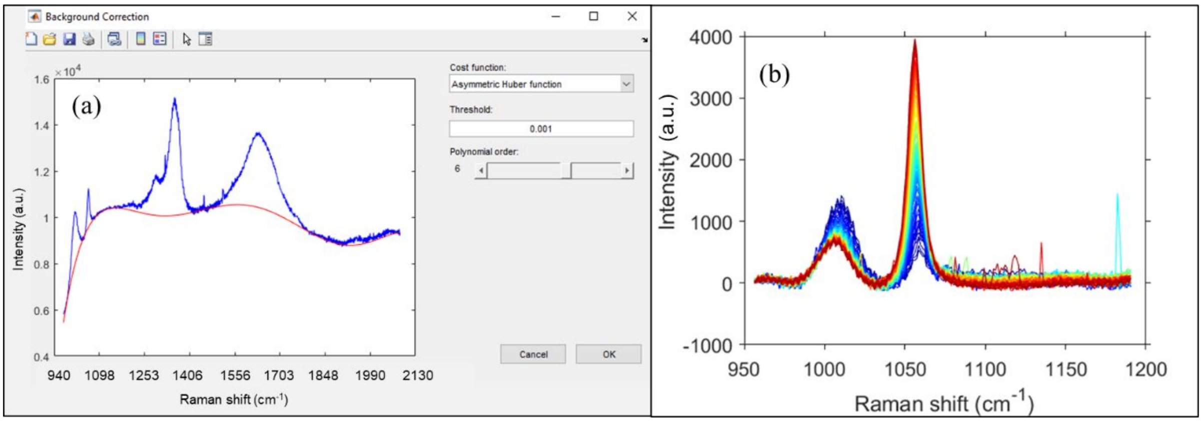

Figure S1. Baseline subtraction process on MATLAB, including (a) the MATLAB interface for baseline subtraction; (b) an example of a set of Raman spectra with baseline subtracted progressing in time from blue to red.

S1.1.2. Chemical Solutions

Solutions are made by dissolving sodium chloride and sodium bicarbonate in HPLC-grade water at a mass ratio 2:1 and molarity of 1.467 mol kg^{-1} and 0.51 mol kg^{-1}, respectively (equivalent to 11.8% *w/w* or 0.118 mass fraction of solute). Both solutes are purchased from Sigma-Aldrich (BioXtra ≥ 99.5%). The carbonic anhydrase (CA) used in this study is from bovine erythrocytes and was purchased from Sigma-Aldrich (C2624). A small amount of CA powder was dissolved in HPLC-grade water to make a 0.015 % *w/w* solution for the low concentration and a 0.077% *w/w* solution for the high concentration of CA. A volume of 3 μL of the CA solution is added to the 40 μL sessile droplet volume directly to allow a time dependent measurement with negligible impact on the ionic solute concentrations. Although the sessile droplet volume for most measurements is set at 40 μL, we also performed measurements on droplet volumes of between 20 and 80 μL to examine the influence of droplet volume on the kinetics of compositional and pH change.

All solution pH values were measured using a pH meter (HANNA Instruments), calibrated with pH 7 solution before each measurement. The pH resolution is 0.1 pH, and the accuracy at 20 °C is ± 0.1. The initial pH of a given solution is always measured at the start of the experiment.

S1.1.3. Gas Phase Conditions

The relative humidity (RH) of the environmental cell is controlled by mixing wet and dry nitrogen flows, each controlled by a mass flow controller (MKS). The RH is recorded by a sensor just before reaching the cell. The typical gas flow rate for measurement is 200 sccm (standard cm^{-3} min^{-1}), although we also performed experiments at lower gas flow rates (0, 50, 100 sccm) to examine the impact of the flow rate on the kinetics of the droplet pH change. A CO_2 gas cylinder is also connected to the cell and the flow rate controlled by another mass flow controller (Fig. S2). The CO_2 concentration was set at 400 ppmV, 5000 ppmV and 25,000 ppmV in a series of measurements to examine the impact of gas phase CO_2 concentration on the final droplet pH.

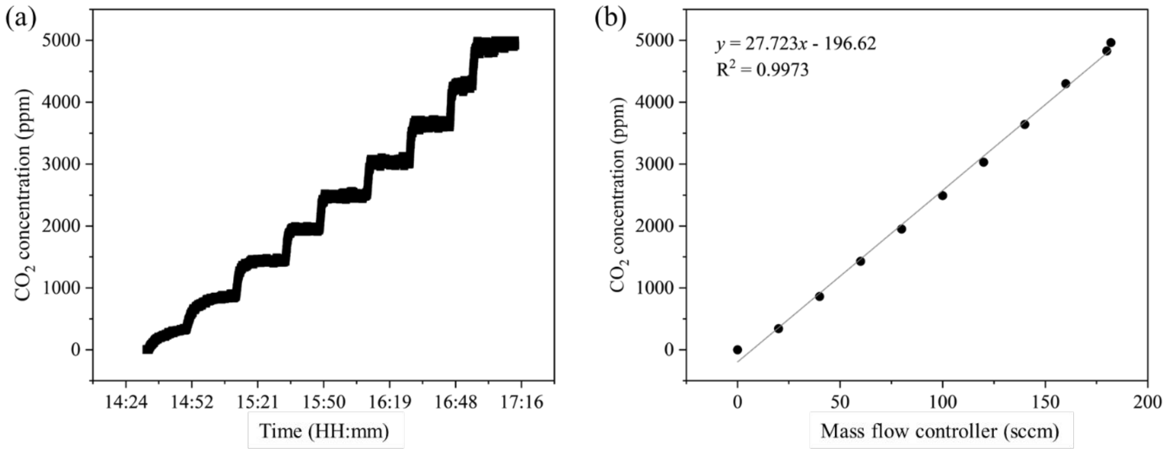

Figure S2. The calibration of the CO_2 metre, including (a) the time taken for the CO_2 to equilibrate, and (b) the equilibrated CO_2 concentration as a function of the gas flow controlled by the mass flow controller.

S1.2. Model and Simulation Details

S1.2.1. The ACCENT Model

ACCENT stands for Atmospheric Composition Change – the European Network of Excellence. The ion concentrations, activity coefficients and equilibrium pH are estimated from the Pitzer activity coefficient model[4] and compared with the experimental results.[5–7] The initial composition of the droplet for model input is 1.47 mol kg^{-1} NaCl and 0.51 mol kg^{-1} $NaHCO_3$ (or 1.98 mol kg^{-1} Na^+, 1.47 mol kg^{-1} Cl^-, 0.51 mol kg^{-1} H^+ and 0.51 mol kg^{-1} CO_3^{2-}).

S1.2.2. Respiratory Aerosol Model (ResAM)

The pH change in the sessile droplet was compared with the Respiratory Aerosol Model (ResAM),[8] a fully Lagrangian model that predicts single droplet thermodynamics and acidity. The activity

coefficients of the involved species are calculated using the Pitzer ion-interaction model.[8–10] The CO_2-Na^+-Cl^- interaction parameters of dos Santos *et al.*[11] were used for the calculation of CO_2 activity coefficients. The calculated pH of $NaHCO_3$ and Na_2CO_3, mentioned above agrees with ACCENT model results within 0.1 pH unit. In addition to the version of published version of Luo *et al.*,[8] the following improvements are made: 1) the chemical reaction of Equations 1 and 2 are solved with and without carbonic anhydrase enzyme; 2) the liquid phase diffusion coefficient of neutral species (H_2O and CO_2) is determined from the viscosity data of aqueous NaCl and Na_2CO_3 and $NaHCO_3$ solutions. These are discussed further below.

i. Catalytic reactions of hydration of CO_2 and dehydration of H_2CO_3

The reaction can be written as (neglecting H_2CO_3 explicitly in the overall reaction):

$$\mathrm{CO_{2(g)}} + \mathrm{H_2O_{(l)}} \overset{k_{41,cat}}{\Longleftrightarrow} \mathrm{H^+_{(aq)}} + \mathrm{HCO^-_{3(aq)}} \quad \text{Eq. S3}$$

The rate of loss of CO_2 due to catalytic reaction of carbonic anhydrase enzyme is defined as:

$$\frac{d\,[CO_2]}{dt} = -\,\vec{k}_{41,cat} \quad \text{Eq. S4}$$

The rate constant for the forward catalytic reaction of carbonic anhydrase enzyme $\vec{k}_{41,cat}$ can be written as:[12]

$$\vec{k}_{41,cat} = k_{cat}\,\frac{[E]}{\frac{K_m}{a_{\mathrm{CO_2}}} + 1} \quad \text{Eq. S5}$$

Here, $[E]$ is the total concentration of enzyme and k_{cat} is the catalytic rate constant in s^{-1}. K_m is the Michaelis–Menten parameter. The loss rate of CO_2 is reduced to 0.5 $k_{cat}[E]$, when the activity of CO_2 equals K_m. The average K_m values were 4 mM for the human erythrocyte carbonic anhydrase (HCA) isoenzyme B (HCA-B) and 9 mM for the HCA-C form.[12]

In this study, the CO_2 concentration (activity) is much lower than 4 mM. Therefore, the forward reaction rate can be approximated by $\vec{k}_{41,cat} \approx \frac{k_{cat}}{K_m}[E]a_{\mathrm{CO_2}}$. The ratio of the catalytic rate coefficient and the Michaelis–Menten parameter (in s^{-1} mol^{-1} kg) can be determined from the present study by fitting to the measurement data:

$$\frac{k_{cat}}{K_m} = 10^{5.45+0.1\times(\mathrm{pH}-7)} \quad \text{Eq. S6}$$

Khalifah[13] reported $\log\left(\frac{k_{cat}}{K_m}\right)$ values of 6.7 and 7.7 for HCA-B and HCA-C, respectively. The present rate coefficient, $\frac{k_{cat}}{K_m}$, is a factor of 20 to 200 smaller than the values reported by Khalifah for HCA-B and HCA-C.

ii. Liquid phase diffusion coefficient

The liquid phase diffusion coefficients of H_2O, CO_2, Na^+, HCO_3^-, CO_3^{2-}, H^+, OH^- are estimated using a method similar to that of Luo *et al.*[8] The concentration dependence of the liquid phase diffusion coefficient of H_2O and CO_2 is estimated based on viscosity data of binary NaCl, Na_2CO_3 and $NaHCO_3$ aqueous solutions. For ions, an additional water activity dependent reduction factor, similar to that used by Luo *et al.*,[8] is introduced (see below).

The concentration dependence of the H_2O diffusion coefficient in pure NaCl solution is given by Schaub *et al.*[14] For $NaHCO_3$/Na_2CO_3 solutions, the concentration dependence of the diffusion coefficient is calculated using the viscosity data for $NaHCO_3$ and $NaHCO_3$ solutions from Ozdemir *et al*:[15]

$$\ln\left\{\frac{D_{H_2O}(m_1,m_2)}{D^0_{\mathrm{H_2O}}}\right\} = \left(0.1946 \times \frac{m_1}{m_1+m_2} + 0.2397 \times \frac{m_2}{m_1+m_2}\right) \times m_{\mathrm{N_a^+}} \quad \text{Eq. S7}$$

Here, m_1, m_2 are the molalities of HCO_3^- and CO_3^{2-} ions, respectively. $D^0_{\mathrm{H_2O}}$ is the temperature-dependent self-diffusion coefficient in water. We use the Vogel–Fulcher–Tammann equation to describe the temperature dependence of $D^0_{\mathrm{H_2O}}$. The parameters (in $m^2\ s^{-1}$) are obtained by fitting the data reported by Krynick *et al*:[16]

$$D^0_{\mathrm{H_2O}} = 3.93 \times 10^{-7} \exp\left(-\frac{1073.4}{T-90.9}\right) \quad \text{Eq. S8}$$

Here, T is the temperature (in K). The H_2O liquid phase diffusion coefficient in a NaCl and $NaHCO_3$/Na_2CO_3 mixture solution can be calculated using the liquid phase diffusion coefficient of NaCl solution D_{NaCl} and the diffusion coefficient of H_2O in the $NaHCO_3$/Na_2CO_3 solution $D_{H_2O}(m_1,m_2)$:

$$D_{\mathrm{H_2O}}(m_{\mathrm{NaCl}}, m_1, m_2) = D^{(1-\alpha x)}_{\mathrm{H_2O,\,NaCl}} \times D^{\alpha x}_{\mathrm{H_2O}}(m_1,m_2) \quad \text{Eq. S9}$$

with $x = \frac{m_{\mathrm{Cl}^-}}{m_{\mathrm{Na}^+}}$ and $\alpha = \exp(0.8 \times (1-x))$, m is the molality (in mol kg^{-1}). m_1 and m_2 are the molality of $NaHCO_3$, Na_2CO_3, respectively. The diffusion coefficient of molecular CO_2 is assumed to be:

$$D_{CO_2}(m_{NaCl}, m_1, m_2) = D_{H_2O}(m_{NaCl}, m_1, m_2) \times \frac{D^0_{CO_2,298\,K}}{D^0_{H_2O,298\,K}} \qquad \text{Eq. S10}$$

$D^0_{H_2O,298\,K}$ and $D^0_{CO_2,298\,K}$ are the liquid phase diffusion coefficients of H_2O and CO_2, respectively, in an infinitely dilute aqueous solution at 298.15 K.

Luo *et al.*[8] reported the diffusion coefficient of ions are slower than those of neutral H_2O molecules. We also introduce a reduction factor f for the diffusion coefficient of ions:

$$D_{ion}(m_{NaCl}, m_1, m_2) = D_{H_2O}(m_{NaCl}, m_1, m_2) \times \frac{D^0_{ion,298\,K}}{D^0_{H_2O,298\,K}} \times f \qquad \text{Eq. S11}$$

Best agreement with the experimental data (Figs. 6,7) is obtained using the following equation for $a_w \geq 0.5$:

$$f = \exp\left(-22.5 \times (1 - a_w) + 31.85 \times (1 - a_w)^2 - 14.679 \times (1 - a_w)^3\right. \qquad \text{Eq. S12}$$

For $a_w > 0.5$, the value $a_w = 0.5$ is used.

A sensitivity simulation is shown in Figure S7 for $f = 1$, i.e., without reduction for the ion diffusion coefficient. The agreement with experimental data for 0 ppmV CO_2 and enzyme is much worse. The simulated pH difference between high and low enzyme concentrations is much larger than the observed values. With reduced f, it becomes partially liquid phase diffusion limited. Using the diffusion coefficient of simulated lung fluid (SLF), it becomes entirely limited by liquid phase diffusion for solutions with enzymes (results not shown), as the organics and proteins in SLF result in a much lower liquid phase diffusion coefficient.[8]

The diffusion coefficients of H_2O, CO_2 and involved ions are given in Table S1. The table also includes diffusion coefficients for the initial solution with 1.467 mol kg^{-1} NaCl and 0.51 mol kg^{-1} $NaHCO_3$, as well as for the solution after substantial evaporation of carbonate, which contains 1.467 mol kg^{-1} NaCl and 0.255 kg^{-1} Na_2CO_3.

The water activity dependence of the liquid phase diffusion in the initial solution (1.467 NaCl + 0.510 $NaHCO_3$) with varying water content is shown in Figure S8. The ion diffusion coefficient is smaller by a factor of 10 compared to that of neutral species, consistent with the findings of Luo *et al.*[8]

Table S1: Liquid phase diffusion coefficients (in unit of 10^{-9} m^2 s^{-1}) in water and in $NaCl/NaHCO_3/Na_2CO_3$ mixture solutions.

	Water, 298.15 K	1.467 NaCl + 0.510 $NaHCO_3$, 293.15 K	1.467 NaCl + 0.255 Na_2CO_3, 293.15 K
H_2O	2.214[17]	0.495	0.544
CO_2	1.91[18]	0.427	0.0.470
H^+	9.311[18]	0.392	0.491
OH^-	5.273[18]	0.222	0.278
Na^+	1.334[18]	0.056	0.070
Cl^-	2.032[18]	0.085	0.107
HCO_3^-	1.185[18]	0.050	0.062
CO_3^{2-}	0.923[18]	0.039	0.049

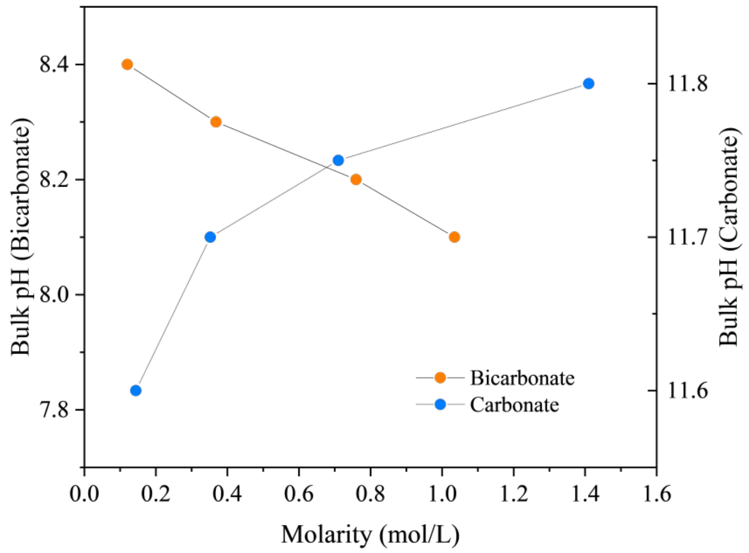

Figure S3. The bulk solution pH measured at different concentrations for aqueous sodium bicarbonate (in orange dots) and sodium carbonate (in blue dots) solutions.

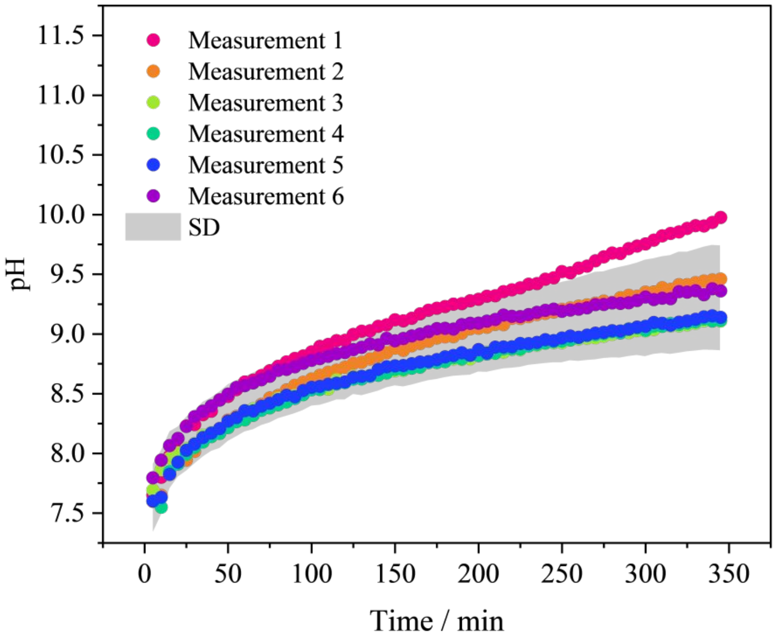

Figure S4. pH profile from six Raman measurements and the standard deviation of a 40 µL droplet, 200 sccm gas flow rate.

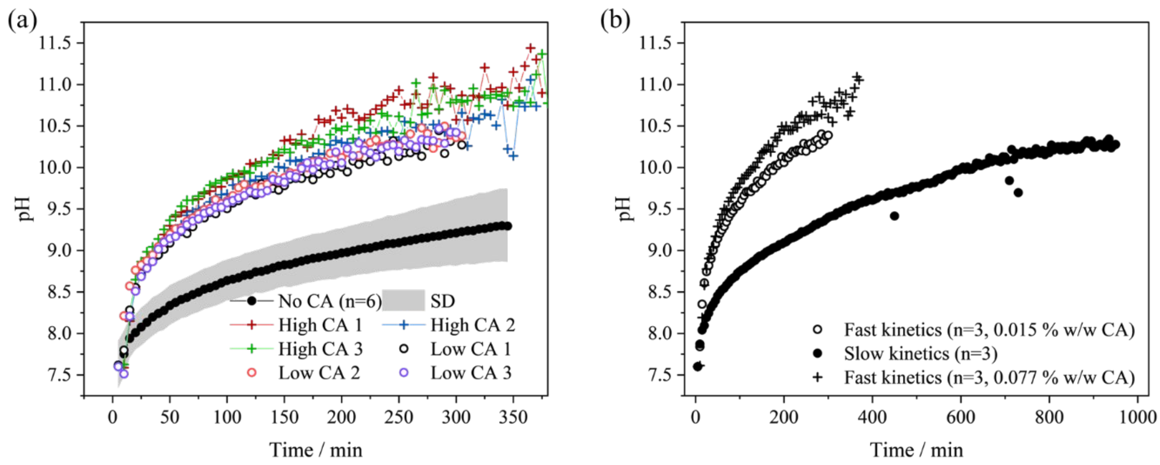

Figure S5. (a) pH profile of measurements including three repetitions of high CA (in cross mark) and low CA (in circle mark) and the average of six repetitions without CA (black circles); (b) average pH profile of longer measurements including three measurements containing 0.015% *w*/*w* CA (low CA), three measurements containing 0.077% *w*/*w* CA (high CA), and three 16-hour measurements with no CA.

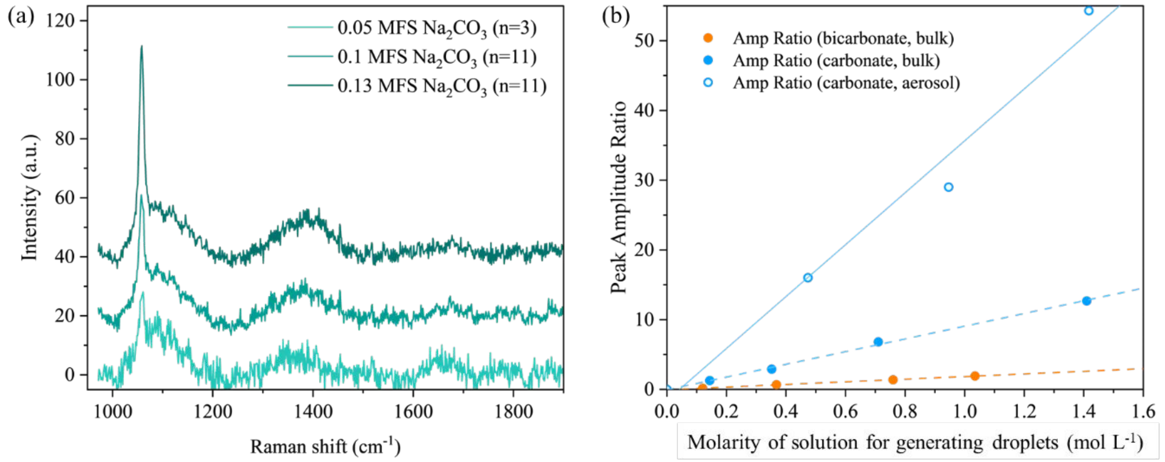

Figure S6. (a) Measured aerosol Raman spectra of pure sodium carbonate solution of varying concentrations (in mass fraction of solute, MFS). (b) Ratio of Raman peaks of bicarbonate (in orange colour) and carbonate (in blue colour) relative to water peak as a function of molarity in bulk (solid dots) and aerosol phase (circles).

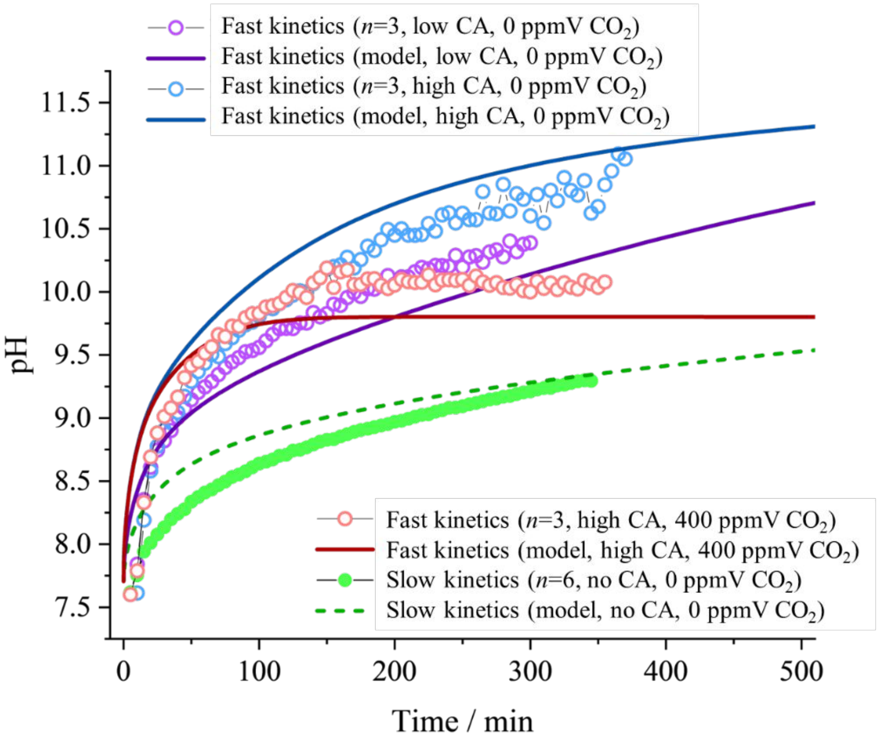

Figure S7. Sensitivity simulation for the diffusion coefficient of ions. This figure is similar to Figure 7 but assumes that ions diffuse in solution like neutral species such as H_2O and CO_2, i.e., the factor f in Equation S12 equals 1. The catalytic rate coefficient k_{cat}/K_m is reduced by 65% to compensate for the

fast liquid phase diffusion. The difference between the two simulated curves with CA enzymes at 0 ppmV CO_2 (blue and pink) becomes larger than the measurement. In Figure 7, with a reduced diffusion coefficient for ions, the system is partially liquid phase diffusion limited and agrees with the measured data.

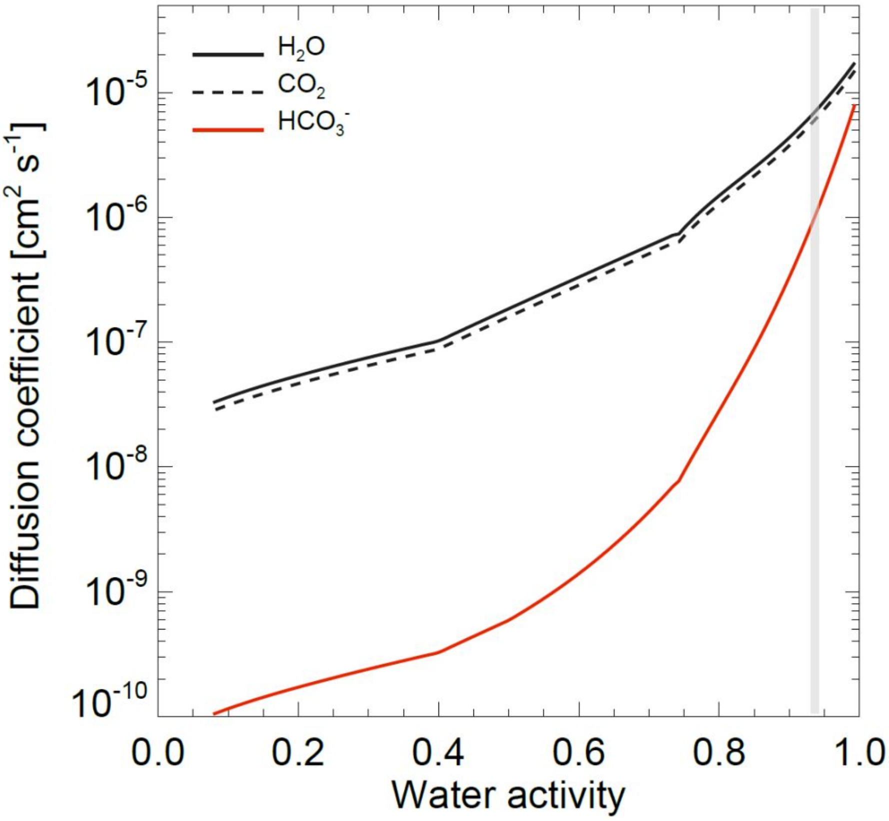

Figure S8. Liquid phase diffusion coefficients of H_2O, CO_2 and bicarbonate ions in the initial composition (1.467 NaCl + 0.510 $NaHCO_3$), excluding H_2O. Different water activities are achieved by either adding water to or removing water from the solution. The vertical bar shows the water activities of the solutions studied in this paper.

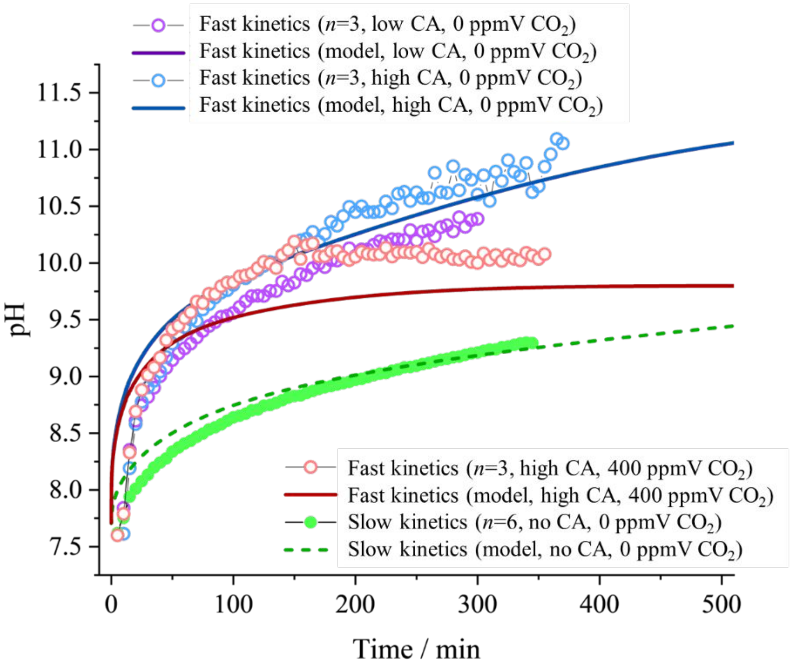

Figure S9. Sensitivity simulation for the catalytic rate coefficient. This figure is similar to Figure 7 but a catalytic rate of Khalifah *et al*.[13] for HCA-B ($\frac{k_{cat}}{K_m}$ = $10^{6.7+0.4\times(\mathrm{pH}-7)}$ s^{-1} kg mol^{-1}) is used, which is a factor 30 – 200 higher than this study, depending on pH.. For 0 ppmV CO_2 with enzyme present, the rate becomes solely diffusion limited, showing no difference between low and high enzyme concentrations. The diffusion coefficient shown in Fig.S8 is reduced by 30% for this sensitivity simulation for the best agreement with measured data.

Table S2: Comparison of simulated pH and CO_2 vapour pressure outputs from the ResAM model, ACCENT model, and the latest update of the ACCENT model: the MarChemSpec (MCS) model,[19] for varying initial concentrations of sodium chloride and sodium bicarbonate solutions. *m* refers to the molality of the compounds/ions, and a_w refers to water activity. Values in bold indicate the uncertainty/limitations of the model simulations at low water activity.

m NaCl	*m* $NaHCO_3$	*m* Na^+	*m* Cl^-	*m* HCO_3^-	a_w		pH				*p*CO_2 atm			
					ResAM	ACCENT	ResAM	ACCENT (25 °C)	MCS (25 °C)	MSC (20 °C)	ResAM	ACCENT (25 °C)	MCS (25 °C)	MSC (20 °C)
0.1467	0.051	0.1977	0.1467	0.051	0.99342	0.99349	8.05	8.03	7.91	7.95	0.02266	0.02318	0.02127	0.01808
1.467	0.51	1.977	1.467	0.51	0.93232	0.93447	7.58	7.65	7.61	7.66	0.41900	0.37780	0.38201	0.32348
4.401	1.53	5.931	4.401	1.53	0.7682	0.78856	**7.05**	**7.15**	**7.72**	**7.77**	**4.76400**	**2.16130**	**2.23777**	**1.88889**
7.335	2.55	9.885	7.335	2.55	0.59978	0.64609	**6.60**	**7.21**	**8.11**	**8.18**	**11.20000**	**3.83000**	**3.71372**	**3.12797**

S2. Statistical Test for Figure 5b

Given the limited sample size of our data, a non-parametric statistical test like Mann-Whiteney U (MWU) test is appropriate for assessing whether the observed differences between droplet sizes are statistically meaningful. Here, we compared the pH values between each droplet volume group – Group 1: 20 µL vs. 40 µL, Group 2: 40 µL vs. 80 µL, Group 3: 20 µL vs. 80 µL) – at 300 and 325 minutes, corresponding to the final pH values across different droplet sizes.

The sample sizes were: 20 µL (n=3), 40 µL (n=6) and 80 µL (n=3). The MWU test results indicate no statistically significant differences between any of the droplet volume pairs. Specifically, at 300 minutes, the p-values were: Group 1 = 0.38, Group 2 = 0.17, and Group 3 = 0.10 (all ≥ 0.05). At 325 minutes, the p-values remained the same: Group 1 = 0.38, Group 2 = 0.17, and Group 3 = 0.10 (all ≥ 0.05). These results suggest that the observed differences in final pH are not statistically significant.

References for Supporting Information

1. Reid, J. P. *et al.* The morphology of aerosol particles consisting of hydrophobic and hydrophilic phases: Hydrocarbons, alcohols and fatty acids as the hydrophobic component. *Physical Chemistry Chemical Physics* **13**, 15559–15572 (2011).
2. Power, R. *et al.* Observation of the binary coalescence and equilibration of micrometer-sized droplets of aqueous aerosol in a single-beam gradient-force optical trap. *Journal of Physical Chemistry A* **116**, 8873–8884 (2012).
3. Mazet. Background correction - File Exchange - MATLAB Central. https://uk.mathworks.com/matlabcentral/fileexchange/27429-background-correction (2024).
4. ACCENT Model. http://www.aim.env.uea.ac.uk/aim/accent4/main.php.
5. Harvie et al. ACCENT Model: The System H+ - NH4+ - Na+ - K+ - Ca2+ - Mg2+ - SO42− - NO3− - Cl− - CO32− - OH− - H2O at 298.15 K. http://www.aim.env.uea.ac.uk/aim/accent4/model.php.
6. Harvie, C. E. & Weare, J. H. The prediction of mineral solubilities in natural waters: the Na□K□Mg□Ca□Cl□SO4□H2O system from zero to high concentration at 25° C. *Geochimica et Cosmochimica Acta* **44**, 981–997 (1980).
7. Harvie, C. E., Møller, N. & Weare, J. H. The prediction of mineral solubilities in natural waters: The Na-K-Mg-Ca-H-Cl-SO4-OH-HCO3-CO3-CO2-H2O system to high ionic strengths at 25°C. *Geochimica et Cosmochimica Acta* **48**, 723–751 (1984).
8. Luo, B. *et al.* Expiratory Aerosol pH: The Overlooked Driver of Airborne Virus Inactivation. *Environmental Science & Technology* **57**, 486–497 (2023).
9. Pitzer, K.S. *Activity Coefficients in Electrolyte Solutions*. (1991).
10. Luo, B., Carslaw, K. S., Peter, T. & Clegg, S. L. Vapour pressures of H2SO4/HNO3/HCI/HBr/H2O solutions to low stratospheric temperatures. *Geophysical Research Letters* **22**, (1995).
11. dos Santos, P. F. *et al.* An improved model for CO2 solubility in aqueous Na+–Cl−–SO42− systems up to 473.15 K and 40 MPa. *Chemical Geology* **582**, 120443 (2021).

12. Gibbons, B. H. & Edsall, J. T. Rate of Hydration of Carbon Dioxide and Dehydration of Carbonic Acid at 25°. *Journal of Biological Chemistry* **238**, 3502–3507 (1963).

13. Khalifah, R. G. The Carbon Dioxide Hydration Activity of Carbonic Anhydrase: I. STOP-FLOW KINETIC STUDIES ON THE NATIVE HUMAN ISOENZYMES B AND C. *Journal of Biological Chemistry* **246**, 2561–2573 (1971).

14. Schaub, A. *et al.* Salt supersaturation as accelerator of influenza A virus inactivation in 1-µl droplets. 2023.12.21.572782 Preprint at https://doi.org/10.1101/2023.12.21.572782 (2024).

15. Ozdemir, O., Çelik, M. S., Nickolov, Z. S. & Miller, J. D. Water structure and its influence on the flotation of carbonate and bicarbonate salts. *Journal of Colloid and Interface Science* **314**, 545–551 (2007).

16. Krynicki, K., Green, C. D. & Sawyer, D. W. Pressure and temperature dependence of self-diffusion in water. *Faraday Discuss. Chem. Soc.* **66**, 199–208 (1978).

17. Yaws, C. L. Chapter 4 - Viscosity of Liquid – Inorganic Compounds. in *Transport Properties of Chemicals and Hydrocarbons (Second Edition)* (ed. Yaws, C. L.) 255–259 (Gulf Publishing Company, Oxford, 2014). doi:10.1016/B978-0-323-28658-9.00004-4.

18. Rumble, J. *CRC Handbook of Chemistry and Physics*. (Taylor & Francis Group, Boca Raton, FL).

19. SCOR Working Group 145 | MARCHEMSPEC: Chemical Speciation Modelling in Seawater to Meet 21st Century Needs. https://marchemspec.org/ (2024).
